# HIV, the gut microbiome and clinical outcomes, a systematic review

**DOI:** 10.1371/journal.pone.0308859

**Published:** 2024-12-09

**Authors:** Rachel Mac Cann, Ellen Newman, Declan Devane, Caroline Sabin, Aoife G. Cotter, Alan Landay, Paul W. O’Toole, Patrick W. Mallon

**Affiliations:** 1 School of Medicine, University College Dublin, Dublin 4, Ireland; 2 Department of Infectious Diseases, St Vincent’s University Hospital, Dublin 4, Ireland; 3 Centre for Experimental Pathogen Host Research (CEPHR), University College Dublin, Dublin 4, Ireland; 4 School of Nursing and Midwifery, National University of Galway, Galway, Ireland; 5 Institute for Global Health, Universitay College London, London, United Kingdom; 6 Department of Infectious Diseases, Mater Misericordiae University Hospital, Dublin 7, Ireland; 7 Department of Internal Medicine, Rush University, Chicago, Illinois, United States of America; 8 School of Microbiology & APC Microbiome Ireland, University College Cork, Cork, Ireland; Kaohsiung Medical University, TAIWAN

## Abstract

**Background:**

Effective antiretroviral therapy (ART) has improved the life expectancy of people with HIV (PWH). However, this population is now experiencing accelerated age‐related comorbidities, contributed to by chronic immune activation and inflammation, with dysbiosis of the gut microbiome also implicated.

**Method:**

We conducted a systematic literature search of PubMed, Embase, Scopus, Cochrane reviews and international conference abstracts for articles that examined for the following non-communicable diseases (NCDs); cardiovascular disease, cancer, frailty, metabolic, bone, renal and neurocognitive disease, in PWH aged >18 years. Studies were included that measured gut microbiome diversity and composition, microbial translocation markers or microbial metabolite markers.

**Results:**

In all, 567 articles were identified and screened of which 87 full‐text articles were assessed for eligibility and 56 were included in the final review. The data suggest a high burden NCD, in particular cardiovascular and metabolic disease in PWH. Alterations in bacterial diversity and structure varied by NCD type, but a general trend in reduced diversity was seen together with alterations in bacterial abundances between different NCD. Lipopolysaccharide was the most commonly investigated marker of microbial translocation across NCD followed by soluble CD14. Short-chain fatty acids, tryptophan and choline metabolites were associated with cardiovascular outcomes and also associated with chronic liver disease (CLD).

**Conclusions:**

This systematic review is the first to summarise the evidence for the association between gut microbiome dysbiosis and NCDs in PWH. Understanding this interaction will provide insights into the pathogenesis of many NCD and help develop novel diagnostic and therapeutic strategies for PWH.

## Introduction

With effective antiretroviral therapy (ART), many people with HIV (PWH) are now achieving a life expectancy approaching that of the general population [1]. Accumulating evidence points to accelerated aging phenotypes in PWH, with increased risk of several age-related non-communicable diseases (NCD) observed in both resource-rich and limited care settings [1–4].

Unresolved chronic inflammation has been linked to the development of NCD in PWH with gut dysbiosis proposed as one of several underlying mechanisms [5]. HIV infection and viral replication induces profound disruption of gut-associated lymphoid tissue (GALT), which can lead to alterations in gut microbial diversity and species richness. Moreover, commensal bacteria crucial for a healthy gut are replaced by ones that contribute to chronic inflammation and immune dysfunction [6–8]. These compositional changes have been described as a HIV-related microbiota index (HMI) [6]. HIV infection also results in increased microbial translocation (MT) and production of microbiota-produced metabolites [9]. MT occurs when the bacteria (or bacterial products) translocate from the gastrointestinal tract into the systemic circulation, where they can drive inflammation. Markers used for MT include lipopolysaccharide (LPS), soluble C14 (sCD14), β- D -Glucan (BDG) and intestinal fatty acid binding protein (I-FABP), a marker of enterocyte damage. These have been associated with several NCD in the general population such as inflammatory bowel disease [10], liver fibrosis [11] and cardiovascular disease (CVD) [12]. Increased MT has also been associated with poorer health outcomes in PWH [13] including progression of disease and mortality [14].

Microbiota-derived metabolites such as short chain fatty acids (SCFA) can also influence gut function and have been implicated in several NCD [15–17]. Choline metabolites can promote inflammation and include trimethylamine-N-oxide (TMAO), produced in the liver, and trimethylamine (TMA), a product exclusively produced by the gut microbiota [18]. TMAO converts macrophages into foam cells which contribute to atherosclerotic plaque formation [19]. Elevated TMAO levels are linked to higher risk of CVD [20, 21], metabolic dysfunction-associated fatty liver disease (MAFLD) [22], obesity [23] and diabetes [24] in the general population. Disturbances in tryptophan metabolism, primarily mediated through the kynurenine pathway (KP) have also been linked to numerous NCD in the general population, including metabolic syndrome [25], CVD [26], schizophrenia, depression and dementia [27], with metabolites of the KP emerging as implicated in development of NCD in PWH [28]. These characteristic changes after HIV infection are seen in those chronically infected, and studies suggest that these changes cannot be fully restored even after successful virological suppression with ART [29, 30].

Together, these findings point to a novel link between the gut microbiome, MT and microbiota-derived metabolites with the risk of NCD in chronically infected PWH. With a growing aging population of PWH come new challenges with the management of age-related comorbidities. Understanding the knowledge gaps linking inflammation, gut dysfunction and aging in PWH is important for improving health outcomes and identifying those at higher risk.

### Objectives

To address this evidence gap, the aim of this systematic review is to summarise the current understanding of the impact of gut microbiome alterations on inflammation and NCD in PWH.

## Methods

### Protocol and registration

The full protocol, including analysis methods and inclusion criteria, is registered with PROSPERO, number CRD42022352703. This protocol was updated on 10/05/2024 to extend the search timeline to include published papers from January 2012 to April 2024.

### Search strategy and study selection

**Fig 1:**
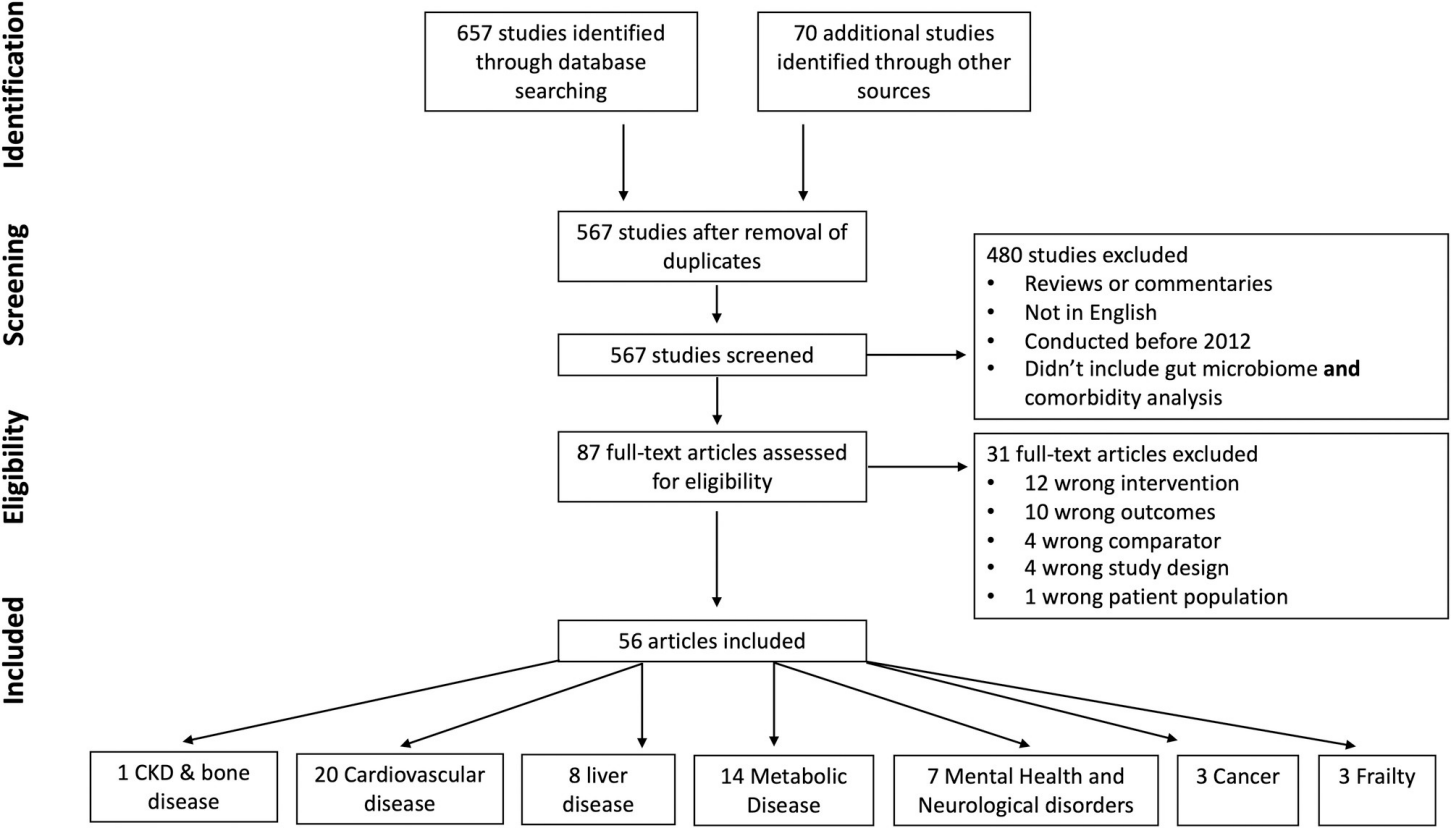
PRISMA flow diagram. Flow chart of study selection process.

Using the Preferred Reporting Items for Systematic Reviews and Meta-analyses (PRISMA) guidelines [31] (S1 Table), we conducted a search of Pubmed, Scopus, Embase and Cochrane Reviews for randomized control trials, cohort, case–control and cross-sectional studies published from 1 January 2012 to 21 April 2024 (S2 Table for data sources and search terms, including Mesh headings and Emtree terms). Reference lists from relevant articles were also screened. Only original articles of adult human studies of the gut microbiome published in English were included (Fig 1). We excluded reviews, commentaries and letters. Two reviewers (RMC and EN) screened all identified titles and abstracts. Selected abstracts underwent full text review and were assessed for eligibility, with all authors agreeing on the final studies for inclusion. In this systematic review, all necessary data were available from the included studies. There were no instances of missing data, so no additional steps were required to address data gaps.

### Quality assessment and data extraction

Extracted data included information on the study authors, year of publication, mean population age, study design, NCD assessed and major findings (NCD association to MT, microbial metabolites and/or gut microbiome compositions). Extraction was conducted using a standardised data extraction form and data were cross-checked for accuracy by the reviewer (RMC) against the source. Quality and risk of bias were assessed using the Joanna Briggs Institute (JBI) Australia tools as appropriate to the study design (see S3–S6 Tables) [32–34]. If a paper included more than one study design (i.e. a cross-sectional analysis and a nested case-control analysis), a JBI score was allocated to each.

## Results

### Study selection

**Fig 2:**
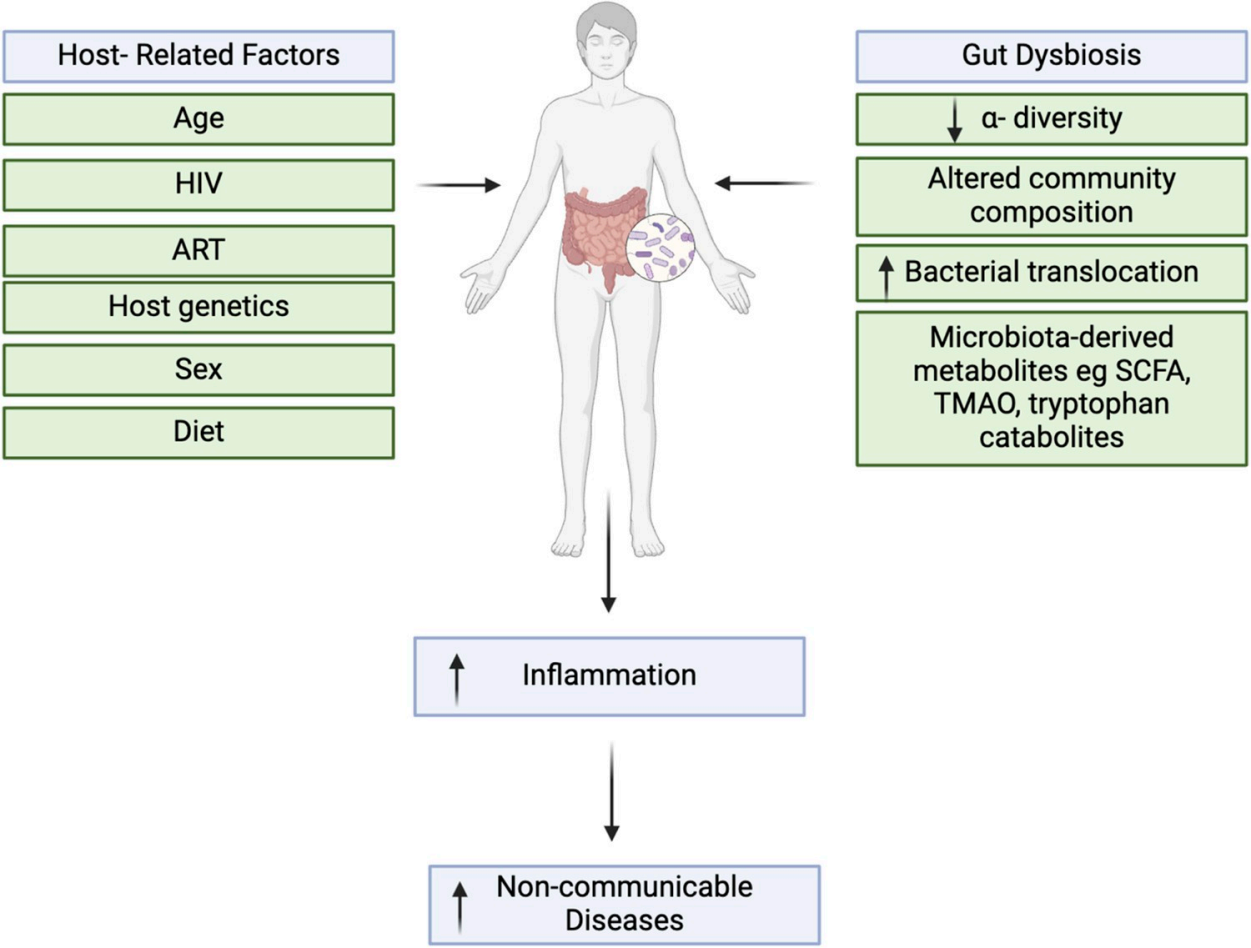
Interrelationships between gut dysbiosis, inflammation and non-communicable diseases (NCDs). The gut microbiota is shaped by various host factors and has a bidirectional relationship with inflammation, and depending on its composition, it can inhibits or stimulate inflammatory pathways. These, in turn, can promote the onset of various inflammatory conditions such as NCDs.

Initial searches identified 727 studies. After removal of duplicates, 567 underwent abstract review with 480 abstracts that did not meet inclusion criteria removed (Fig 2). The remaining 87 full-text articles were assessed for eligibility and 56 were included in the final review. Article exclusion was predominately due to wrong intervention or outcome measured.

### General characteristics of included studies

Of the 56 studies included, the highest number of studies were from the USA (n = 30), followed by Denmark and/or Norway (n = 10), Spain (n = 4) and Thailand (n = 3) with the remainder from Italy, China (all N = 2), the UK, Mexico, Australia, Canada and Germany (all N = 1). Most studies (n = 50) included fewer than 500 participants and the mean/median age ranged from 33 to 61 years. Eight studies were conducted in women only and five were conducted in men only. The proportion of males in the 43 mixed-sex studies ranged from 44%-96%. Thirty-six studies (63%) involved virally suppressed populations, ten (17.5%) included mixed populations of PWH with either suppressed or detectable viral loads, and five studies (8%) focused on PWH with detectable viral loads. Five studies (8%) did not specify the viral load status of their cohort.

Among gut microbiome measurements, 25 studies performed 16S rRNA gene amplicon sequencing on faecal bacterial DNA and three studies performed shotgun sequencing for entire community bacterial DNA. The most commonly measured MT markers were sCD14, LPS and LPS-binding protein (LPB). Twelve studies assessed the role of choline metabolites and six studies explored tryptophan metabolism.

### Quality assessment

Using the JBI tool, 33 of 40 cross-sectional studies scored at least 6 out of possible 8 points, indicating good reliability and relevance, while the two RCTs scored poorly, scoring 4 and 6 out of a possible 12 points, respectively (S3–S6 Tables). This was largely explained by lack of detail on the RCT design and follow-up as these studies reported post-hoc analyses of larger clinical trials. Major sources of potential bias included poor reporting of inclusion and exclusion criteria and lack of consideration for potential confounding from factors such as diet, sex or socio-economic status.

The chosen papers dealt with one of seven NCD; CVD, metabolic disease, CLD, bone disease, cancer, frailty and neuro-psychiatric disease. Associations between gut microbiome and each specific NCD are reported with the following format; first describing gut microbiome diversity and bacterial composition, then MT marker associations and finally data on choline and KP metabolites with the NCD.

### Cardiovascular disease

The risk of CVD in PWH is increased beyond that explained by traditional risk factors alone, with an approximate two-fold increased risk of myocardial infarction (MI) in PWH [35]. CVD accounts for the largest number of studies included in this review (N = 20, Table 1).

**Table 1:** Cardiovascular disease.

	Author, year	Study design	Age	% male	HIV Virological Control	NCD Examined	Main findings
1	Kelesidis 2012, USA	Longitudinal analysis of cohort study (A5078), 1. PWH (n = 55) and 2. HIV negative (n = 36)	Years (median, IQR): 41 (38–45), HIV- 40 (36–45)	1. 96%, 2. 89%	84% undetectable/ supressed viral load	CIMT	Serum sCD14 and LPS were associated with the progression of subclinical atherosclerosis.
2	Blodget 2012, USA	Sub-study of RCT (A5152s) (n = 75) 1. Baseline and 2. Week 24, and cross-sectional analysis of Indiana University Cohort study (n = 85). 3. On ART, 4. Not on ART	Years (mean, SD) 1. 35¬±8 2. 35¬±8 3. 40¬±8 3. 38¬±11	1. 92% 2. 94% 3. 63% 4. 79%	1. 0%, 2. 92%, 3. 84%, 4. 11% undetectable/ suppressed viral load	Endothelial function, assessed by ultrasonic measurement of brachial artery FMD 60 seconds after lower forearm cuff release expressed as a % change	No significant correlation between brachial FMD and either sCD14 or LPS across groups in A5152s study. Negative correlation between LPS and brachial FMD, with a progressive step-wise decrease in median % FMD across increasing tertiles of LPS levels om Indiana cohort study.
3	Manner 2013 Norway	Cohort study, PWH (n = 42) and HIV negative (n = 15)	Years (median, IQR) PWH 42 (32–46), HIV- 40 (36–45)	80%	44000 (3550–165000) copies/mL (median, IQR)	BP at two visits	Plasma levels of LPS correlated with sCD14 in PWH. LPS and sCD14 were strongly correlated in patients with HTN with a stepwise increase in BP across the tertiles of LPS and sCD14.
4	Qi 2018 Australia	Cohort study, 1. PWH (n = 520) and 2. HIV negative (n = 217) at two time points, median follow up 7 years	Years (median, IQR) 1. 44 (40.5, 42), 2. 44.5 (41.5–50.5)	1.44%, 2.50%	women- 160 (80–4700), Men 40 (40–1280) copies/mL, (median, IQR)	CAP measurement by carotid ultrasound; sCD14, sCD163, galectin-3, Gal-3 binding protein, CRP, IL-6	Tryptophan, kynurenic acid, and KTR were higher in PWH and correlated with serum sCD14, Gal-3. Higher plasma tryptophan was significantly associated with a lower risk of CAP while higher plasma kynurenic acid and KTR were significantly associated with an increased risk of CAP.
5	Sinha 2015 USA	Case control study, PWH (n = 82) and HIV- (n = 20)	Years (mean, SD) PWH 50.6 +- 9.6, HIV- 48.9 +- 12.4	94%	75.9% undetectable/ suppressed viral load	CIMT	PWH had similar TMAO levels as uninfected individuals with CAD. With increasing TMAO and carnitine, there was a mild association with increased mean CIMT.
6	Srinivasa 2015 USA & France	Cross sectional study, 1.PWH (n = 155) and2. HIV–(n = 67)	Years (mean, SD) PWH 47 ± 7, HIV- 46 ¬± 7	1.61%, 2.58%	86% undetectable/ suppressed viral load	CTA imaging for assessment of CAP; VAT and SAT by CT scan; Dietary intake collected through a 4-day food record.	PWH had a significantly higher prevalence of CAP and number of total CAP segments. In PWH, serum TMA was significantly and positively associated with calcium score, number of total CAP, number of calcified CAP, calcium plaque volume and calcium plaque mass. TMA levels inversely correlated with HDL and were positively correlated with LPS.
7	Haissman 2016, Norway	Cross sectional study, 1. PWH without CHD (n = 105), 2. HIV- without CHD (n = 105), And Case-control study 3. PWH with first-time MI (n = 55), 4. HIV- controls (n = 182)	Years (mean, SD) PWH 46 +- 8, HIV—47 +-9	1. 89%, 2. 89%; 3. 91% 4. 92%	90% undetectable/ suppressed viral load	Myocardial perfusion scintigraphy, CACS, CIMT; The Framingham risk score; Measurement of Cholesterol, triglycerides, and glucose	No difference in plasma TMAO or choline between groups with first-time MI, but betaine was lower in HIV-infected person;. 18% of PWH had MPD vs. 0% among HIV- and MPD was associated with elevated TMA.
8	Elliott-Miller 2016, USA	Case control study of MACS cohort (n = 102), Cases = CAP > 50% in 1 or more coronary segments, (n = 51). Controls = No CAP on CCTA, (n = 51)	Years (median, IQR) cases 55 (51–58), controls 54 (51–59)	100%	80% undetectable/ suppressed viral load	Non-contrast cardiac CT and CCTA to define presence/ absence of atherosclerosis.	TMAO levels showed an inverted U shaped association with coronary artery stenosis in PWH, not controls.
9	Knudsen 2016, Denmark & Norway	Cross sectional study- SHADE, PWH (n = 94)	Years (mean, SD) 49.6 +-1.0	53%	100% undetectable/ suppressed viral load	MBF reserve determined by 82Rb PET/CT at rest and during adenosine-induced stress; Framingham risk score	No association between TMAO and myocardial perfusion, left ventricular ejection fraction, age or CAC score as assessed by 82Rb PET/CT.
10	Haissman 2017, Norway	Cross sectional study, PWH on cART n = 50, PWH not on cART n = 50	Years (median, IQR) on ART 43 (36–48), not an ART 41 (33–46)	88%	1. Untreated PWH 23,026 (5517–90,321), 2. Treated PWH 19 (19–20), copies/mL (median, IQR)	Platelet function was determined by whole-blood multiple electrode impedance aggregometry.	Elevated ratios of TMAO/betaine and TMAO/carnitine found in PWH on ART.
11	Shan 2018, USA	Cohort study (WIHS and MACS cohorts),1. PWH n = 520, 2.HIV- n = 217	Years (median, IQR) PWH women 42 (38–46), men 46 (43–50), HIV- women 42 (38–47), men 47 (45–54)	1.44%, 2. 50%	1. women- 160 (80–4700), 2. Men 40 (40–1280) copies/mL, (median, IQR)	CAP measurement by carotid ultrasound; Serum measurement of sCD14, sCD163, galectin-3, galectin-3 binding protein, CRP, and IL-6. Plasma measurement of TMAO and KP	Higher plasma TMAO was significantly associated with an increased risk of CAP in PWH; TMAO was significantly positively correlated with plasma KTR and sCD14;
12	Kehrmann 2019, Germany	Cross sectional study of PWH with CHD (n = 30) and without CHD (n = 30)	Years (mean, SD) CHD + 54.1± 11.2, CHD—51.6 ¬± 10.7	93%	96% undetectable/ suppressed viral load	CHD+ defined by: (1) diagnosis of CHD by positive heart catheter examination; (2) history of MI; and (3) history of CHD+	Lower α-diversity in CHD+ compared to CHD-. CHD- co-occurrence network defined by abundant genera *Bacteroides* and *Prevotella*; CHD+ co-occurrence network defined by a *Prevotella* and a *Ruminococcaceae* abundance. The Prevotella-rich cluster was largely composed of MSM (97%), whereas the Bacteroides-rich cluster comprised both MSM (45%) and heterosexual individuals (55%). Higher TMAO was associated with a higher relative abundance of 4 OTUs, belonging to the genera *Phascolarctobacterium*, *Desulfovibrio*, *Sutterella*, and *Faecalibacterium*.
13	Sinha 2019, USA	A cross sectional analysis of 1. the SCOPE cohort (n = 162) and a case-control analysis of the CNICS cohort- 2. PWH (n = 36 cases (MI), 3. n = 69 controls (no MI))	Years (median, IQR) 1.49 (42–55), 2. 50 (47–58), 3. 49(46–57)	1.91%, 2.78%, 3.77%	75 (50–982) copies/mL (median, IQR)	1. total cholesterol, HDL, lLDL, triglycerides, Il-6, hs-CRP and d-dimer measurements; 2 & 3: CIMT measurements at baseline and at the end of the study (median time 3.1 yrs)	In the SCOPE cohort- carnitine was strongly associated with baseline CAP but both betaine and carnitine were significantly associated with progression of CIMT. IL-6 and D-dimer were associated with atherosclerosis but not with carnitine or betaine. In the CNICS cohort- higher carnitine levels carried increased risk of MI.
14	Montrucchio 2020, Italy	Cross sectional analysis of cohort study of PWH and HIV–(n = 175) and a probiotic intervention pilot study (n = 25)	Years (median, IQR) 50 (44–55)	79.40%	90.7% undetectable/ suppressed viral load	CIMT; Framingham; D:A:;, ASCVD/AHA/ACC and CUORE scores;	Higher TMAO levels associated with higher CIMT;
15	El-Far 2021, USA	Cross sectional study, PWH (n = 79) and HIV- (n = 49)	Years (mean, SD) PWH Plaque—52.48 +- 6.27, PWH Plaque + 55.55 +-6.54, HIV- Plaque + 55.81 +-7.47, HIV- Plaque- 53.05 +- 6.39	100%	99.9% undetectable/ suppressed viral load	Non-contrast cardiac CT and CTA to define presence/ absence of atherosclerosis; Inflammatory markers measured included six IL-32 isoforms (α, β, γ, D, ϵ and θ), IL-18, IL-β, IL-10, TNF-α and IL-6	All IL-32 isoforms were higher in PWH; IL-32D and IL-32θ isoforms were further upregulated in HIV+ CAD+ compared to HIV+ CAD-; Plasma LBP in PWH correlated with expression of IL-32D, IL-32α, β, and ϵ, IL-18, TNF- α-and IL-6; Increased expression of IL-32 isoforms in PWH with CAP was associated with increased abundance of *Rothia* and *Eggerthella* species) and lower abundance of the SCFA caproic acid,
16	Colaco 2021, USA	Cross sectional study of PWH 1. with DD (n = 94), 2. NDD (n = 101)	Years (mean, SD) 1. 58.0¬±8.1 2. 52.5¬±5.7	71%	20 (20–40) copies/mL, (median, IQR)	ECHO; cardiac MRI, plasma measurement of Choline metabolites	TMAO and choline were higher in the DD+ group and TMAO was associated with ECHO DD indexes; TMAO and betaine were associated with measures of myocardial fibrosis and TMAO was correlated with NT‐proBNP, troponin‐I, galectin‐3, GDF‐15, IL‐6 and sCD14.
17	Wang 2022, USA	A cross-sectional study of the WIHS Cohort of PWH and HIV-, n = 361 (264 plaque- and 97 plaque+), and a longitudinal observational analysis of the same cohort (n = 112)	Years (median, IQR) plaque—52 (46–57) plaque + 57 (52–62)	0%	74.7% undetectable/ suppressed viral load	Carotid artery ultrasound; Plasma metabolomic/ lipidomic profiling	*Fusobacterium* was associated with CAP, diastolic BP, plasma lipids and the metabolites lysophosphatidylcholines, lysophosphatidylethanolamines and diglycerides; *Proteus* was also associated with CAP; *Odoribacter* was inversely associated with CAP and systolic BP; *Adlercreutzia* had a moderate inverse correlation with HbA1c. Baseline plasma gut microbiota-related lipid profiles were significantly associated with incident CAP over 7 years.
18	Wang 2023, USA	A cross-sectional study of the WIHS Cohort of 1. CAP+ (n = 84) and 2. CAP- (n = 236)	Years (median, IQR) 1. 57 (53–62) 2. 51(46–56)	0%	74.6% undetectable/ suppressed viral load	Carotid artery ultrasound; Plasma metabolomic/ lipidomic profiling	CAP was associated with enriched *Fusobacterium nucleatum* and depleted *Roseburia hominis*,* Roseburia inulinivorans*,* Odoribacter splanchnicus*, *Clostridium saccharolyticum*, and *Johnsonella ignava* *Fusobacterium nucleatum* was alo associated serum proteomic markers–eg CXCL9 CAP-associated species were correlated with several plasma metabolites, including the microbial metabolite imidazole-propionate (ImP)
19	Luo 2023, USA	A cross-sectional study of the WIHS Cohort of (n = 361) women with CAP		0%	1. Without plaque 71.0 (36.5, 1,912.5), 2. With plaque 43.0 (33.50, 224.0) copies/mL, (median, IQR)	Carotid artery ultrasound; KP metabolites measured by high performance liquid chromatography.	KYNA and KYNA/TRP were positively associated with plaque, indole-3-propionate (IPA) and IPA/KYNA were inversely associated with plaque. Five gut bacterial genera were positively associated with IPA including *Roseburia* spp., *Eubacterium* spp., *Lachnospira* spp., and *Coprobacter* spp.; An IPA-associated-bacteria score was inversely associated with plaque
20	Peters 2023, USA	A cross-sectional study of the WIHS Cohort of (n = 197) post-menopausal women with CAP	Years (median, IQR) 58 (54, 61)	0%	1.00 (1.00, 20.00), copies/mL, (median, IQR)	Carotid artery ultrasound; Sex steroid hormones and sex hormone binding globulin (SHBG	Higher α-diversity was associated with DHEA-S:DHEA ratio, E2, E1, free E2, E1-S, the E1:4-dione ratio, and the E2:testosterone ratio. Estrogens were positively associated with *Alistipes*, *Collinsella*, Erysipelotrichia, and Clostridia spp; Androgens were positively associated with Actinomyces, Erysipelotrichia, and Clostridia spp; Adrenal precursors were positively associated with *Prevotella*, Gammaproteobacteria, *Actinomyces*, and *Megasphaera spp*

**Abbreviations**: ART, antiretroviral therapy; ASCVD, Atherosclerotic Cardiovascular Disease; BMI, body mass index; BNP, brain natriuretic peptide, BP, Blood Pressure; CAC, coronary artery calcium; CAP, Carotid artery plaque; CIMT, Carotid intima media thickness; CHD, coronary heart disease; CCTA, Cardiac CT angiography; CT, computed tomography; CVD, cardiovascular disease; CVS, cardiovascular system; DD, Diastolic dysfunction; ECHO, Echocardiography; FMD, flow-mediated dilation; FRS, Framingham Risk Score; HDL, high‐density lipoprotein; hsCRP, high‐sensitivity C‐reactive protein; IL-6, interleukin‐6; KTR, Kynurenine to Tryptophan Ratio; MBF, Myocardial blood flow; MI, myocardial infarction; MLWH, men living with HIV; MPD, myocardial perfusion defects; MRI, magnetic resonance imaging; MSM, men who have sex with men; NDD, No diastolic dysfunction; sCD14, soluble CD14; SAT, subcutaneous adipose tissue; SCFA, short-chain fatty acid; VAT, visceral abdominal tissue; WLWH, women living with HIV; PI, protease inhibitors; PWH, people living with HIV; RR, risk ratio; WIHS, Women’s Interagency HIV Study.

Several studies explored diversity and composition changes of the gut microbiome with CVD. Reduced α-diversity was observed in PWH with both clinical CVD (n = 60) and subclinical atherosclerosis [36, 37], but no changes were seen in those with carotid artery plaque (CAP) in the 2022 and 2023 studies of the Women’s Interagency HIV Study (WIHS) cohort [38, 39]. Both studies identified an association between CAP and enrichment of *Fusobacterium nucleatum* alongside depleted levels of *Odoribacter*, with *Fusobacterium* abundance correlating with various plasma lipids and metabolites as well as incidence CAP over seven years [38]. The microbial metabolite imidazole-propionate (ImP) was also positively associated with plaque and several pro-inflammatory markers which they suggest may be related to host immune activation and inflammation. A subsequent study of the WIHS cohort in 2024 found that several sex hormones (oestrogens, androgens and adrenal precursors) tended to associate with lower odds of CAP [40]. These sex hormones were also associated with some bacterial species, which were themselves correlated with CAP. This supports hormonal mechanisms of elevated cardiovascular risk in postmenopausal women with HIV (WWHIV).

in contrast, a study of a predominantly male cohort identified an abundance of *Prevotella* and *Bacteroides* in PWH with CVD, with the *Prevotella*-rich cluster comprising mainly men who have sex with men (MSM) (97%). In addition, a whole genome sequencing study of (n = 129) men with and without HIV found both *Rothia mucilaginosa* and an *Eggerthella unclassified* were enriched in those with subclinical atherosclerosis. They also describe an association between specific IL-32 isoforms and CVD and suggest that these are driven by lower levels of the SCFA caproic acid. Together, these three studies demonstrate a possible relationship between altered bacterial diversity and composition with CVD outcomes in PWH. Further studies involving both male and female participants are needed to determine the influence of sex on these alterations.

Three studies examined the association of the MT markers sCD14 and LPS with CVD outcomes in PWH, including progression of atherosclerosis [41], endothelial dysfunction [42] and hypertension [43]. These associations mirror those in the general population, suggesting that sCD14 and LPS may influence CVD outcomes in PWH, either directly or indirectly.

When reviewing the impact of gut-microbiota derived metabolites on CVD outcomes, the largest body of work (10 studies) has explored the association of choline metabolites with CVD in PWH. Most studied TMAO and its precursors, betaine and carnitine, which were linked with increased mean CIMT and progression of CIMT in PWH [44–46]. Less consistent associations between TMAO and coronary artery plaque (CAP were reported, with one study observing an increased risk (RR 1.25) of CAP in PWH (n = 520) with higher plasma TMAO levels and a smaller study (n = 100) reporting an inverted U-shaped association between TMAO and CAP among men with HIV [47]. Similarly, an increase in another choline precursor, TMA, was found to be significantly associated with several markers of calcification and plaque in 155 PWH. Furthermore, higher TMA was associated with both lower HDL and higher levels of LPS [48].

Although these findings suggest that TMAO is associated with atherosclerotic disease, it was not found to be associated with increased risk of MI in a study of (n = 105) PWH or in first-time MI (n = 237), but was found to be elevated in PWH with silent ischemia [49, 52]. Further work using 82Rb PET/CT found no association between TMAO and myocardial perfusion, left ventricular ejection fraction or CAC score in 94 PWH [50]. Conversely, a multisite cross-sectional study (CHART-HIV) discovered higher TMAO and choline levels in PWH with diastolic dysfunction. TMAO and betaine were also significantly associated with myocardial fibrosis measures, and elevated TMAO correlated with cardiac and MT markers like sCD14, NT‐proBNP, troponin‐I, galectin‐3, GDF‐15, and IL‐6 [51]. These findings suggest that, although elevated TMAO and TMA are linked to atherosclerosis and cardiovascular inflammation, further research in larger studies is needed to confirm if this association translates into differences in clinical outcome.

Recently, attention has turned to exploration of the KP and CVD outcomes. One study of the WIHS and MACS cohorts (n = 737) found that PWH exhibit lower tryptophan and higher KTR than those without HIV [52]. Higher plasma kynurenic acid (KA) and KTR significantly associate with an increased risk of CAP. Another study conducted in just the WIHS cohort (n = 361), showed that plasma KA and KA/TRP ratio were positively associated with CAP [53]. Additionally, indole-3-propionate (IPA) and IPA/KA ratio were inversely associated with CAP independent of HIV status. While these studies suggest that elevated TMAO and KP-related metabolites are associated with CVD, data showing if these associations translate to clinical outcomes is limited. It is likely that a combination of metabolites may influence CVD outcomes in PWH and further research in the area is warranted.

### Metabolic disease

PWH on successful ART have an increased risk of developing obesity, insulin resistance and metabolic syndrome (MetS), independent of demographic and lifestyle factors [6]. Gut dysbiosis and chronic systemic inflammation are common features of PWH with metabolic changes [54]. Fourteen studies examining these components of metabolic disease were included (Table 2).

**Table 2:** Metabolic disease.

	Author, year	Study design	Age	% male	HIV Virological Control	NCD Examined	Main findings
1	Timmons 2014, USA	1. Sub-study of RCT (ACTG 5152s) study (n = 82) PWH 2. Cross-sectional analysis of Indiana University Cohort study, PWH not on ART (n = 47), 3. IU study PWH on ART (n = 49)	Years (mean, SD) 5152s cohort: 1. 35.6¬±8.3 2. Not on ART: 37.0¬±11.5 3. On ART: 40.3¬±8.1	1. 92% 2. 81% 3. 65%	1. 85113.80 ± 123468.52 2. 89.13 ± 151.86 3. 707.95 ± 1173.68 4. 16595.87 ± 38213.40 (mean, SD)	Lipids, lipoproteins, glucose, insulin, Insulin sensitivity was estimated by HOMA-IR. DXA scans at baseline and week 48 in ACTG 5142.	LPS correlated positively with HOMA-IR and triglycerides, particularly in ART naïve. A negative correlation was seen between sCD14 and HDL cholesterol, particularly ART naïve. A negative correlation was seen between sCD14 and trunk fat, limb fat and lean mass by DXA.
2	Villanueva-Millan 2019, Spain	Cross sectional study and a nested case control study of 1.PWH with MS (n = 11) and2. without MS (n = 40)	Years (mean, SD) 1. 48.38±‚ 0.89 2. 52.30¬±1.1	66%	100% undetectable/ suppressed viral load	MS as classified by the NCEP-ATP III Fasting plasma levels of glucose, triglycerides, total cholesterol, LDL, HDL, AST, and ALT, insulin, IL-6, PAI-1 and MCP-1. HOMA-IR for insulin resistance	No differences were found in α-diversity, LBP or sCD14 between MS- and MS+ patients. In MS group, found reduction of several species including *Eubacterium eligens*, *Faecalibacterium prausnitzii*, *Roseburia intestinalis*, *Roseburia inulinivorans*, *Ruminococcus flavefaciens*, *Subdoligranulum s*p, *Sutterella wadsworthensis* and the *Bifidobacterium* genus of the *Actinobacteria* phylum. HIV+MS+ showed higher PAI-1, triglycerides-to-HDL-ratio compared to HIV+MS- The abundance of R. flavefaciens was negatively correlated with PAI-1 and LDL.
3	Armstrong 2021, USA	Cross sectional study of PWH and HIV negative, 1. HIV + MSM untreated- (n = 14) 2. HIV+ MSM, treated (n = 20) 3. HIV+ MSM, treated, with Lipodystrophy- (n = 25) 4. HIV- MSW (n = 22) 5. HIV—MSM- (n = 32)	Years (median, IQR) 1. 34 (26.5–40.3) 2. 46 (42.8–50.5) 3. 60 (54–64) 4. 33 (27.3–38.5) 5. 34 (29.8–44.5)	100%	1.101,400 (20,300–292,514) 2.20 (0–20) 3.0 (0–20) copies/mL, 4.NA 5.NA (median, IQR)	Untargeted metabolomics; Serum analysis of fasting triglycerides, glucose, insulin, LDL, HDL, leptin, and adiponectin and inflammatory cytokines. Diet History Questionnaire II	VSURF selected immune markers that were positively correlated with the metabolic disease score included LBP, ICAM-1, IL-16, IL-12, and GM-CSF. LBP in turn correlated with other markers of systemic inflammation, a loss of beneficial microbes such as butyrate-producing bacteria, and a higher BMI
4	Amador-Lara 2022, Mexico	Cross sectional study of PWH (n = 60)	Years (mean, SD) MS +42.8 ± 10.1, MS—39.4 ± 11.4	76%	92.8% undetectable/ suppressed viral load	Caloric intake measurement Serum analysis of fasting triglycerides, glucose, insulin, LDL, HDL, leptin, HbA1c and adiponectin and inflammatory cytokines. Triglycerides/HDL index, > 3 = predictor of cardiovascular risk HOMA-IR, VAI, FLI, ASCVD FINDRSIC, calculated	Decreased α- diversity in MS+ with a predominance of *Prevotella*, higher abundance of phylum *Bacteroidetes*, *Proteobacteria and Fusobacteria* and a significant decrease in SCFA‐producing bacteria. Higher serum levels of hsCRP in MS+. Higher scores in the HOMA-IR, VAI, FLI, TG/HDL ratio and FINDRISC scale in MS group compared to MS-. Genes related with insulin signalling pathway were significantly increased in MS-.
5	Gelpi 2020, Denmark & Norway	Case control study of 1. PWH MSM (n = 281), 2. PWH non-MSM (n = 124), 3. HIV- MSM (n = 43), 4. HIV- (n = 68)	Years (mean, SD) 1. 53.2 (11.3), 2. 53.4 (10.4), 3. 40.1 (9.6) 4. 60.6 (9.1),	1. 100%, 2. 48.4%, 3. 100% 4. 66.2%,	95.2% undetectable/ suppressed viral load	Non-fasting serum HDL- C, triglycerides, and glucose. CT measures of SAT and VAT.	Identified HMI consisting of lower diversity and increased relative abundance of *Gammaproteobacteria* and *Desulfovibrionaceae* and decrease in several *Clostridia* species. The HMI was positively correlated with sCD14 and LBP and associated with excess risk of MS, hypertriglyceridemia, diabetes, and hypertension A 1-unit increase in α-diversity was associated with a 28% reduced risk of MS. A history of severe immunodeficiency was associated with a 5-fold excess risk between HMI and MS. It also modified the association between the HMI and a 30-cm2 larger VAT area.
6	Gelpi 2022, Denmark	Cross sectional study of (n = 383) PWH	Years (median, IQR) 52 (46.1‚ 61.0)	84.10%	95.3% undetectable/ suppressed viral load	Demographics and lifestyle questionnaires. CT measures of SAT and VAT.	The HMI was associated with higher KTR, QKR and lower KA and Trp concentrations, driven by increase in the family *Desulfovibrionaceae* and genus *Eisenbergiella* and reduction in the *Lachnospiraceae* CAG 56 and *Coprococcus* 2. KTR was associated with higher VAT-to-SAT ratio and larger VAT area. LBP levels were associated with higher levels of KTR and QKR and associated with larger VAT and SAT areas.
7	Gogokhia 2020, USA	Cross sectional study of (n = 35) PWH	Years (mean, SD) 1. visceral obesity- 51.0 (5.9) 2. general obesity- 45.2 (6.6) 3. lean- 45.7 (7.9	1. 86.7% 2. 0% 3. 90.9%	1. 93.3%, 2. 66.7% 3. 100% undetectable/ suppressed viral load	Serum measurement of hsCRP, adiponectin, leptin, IL-6, sCD14 and MCP-1. CT measures of SAT and VAT.	Higher α-diversity index in participants with lean body type, compared with visceral and general obesity. α-diversity was negatively correlated with VAT area, waist/hip ratio and sCD14. Increased abundance of family *Prevotellaceae*, genus *Erysipelatoclostridium* and *Bacteroides* in the lean group. Increased abundance of family *Bacteroidaceae* and reduction in *Flavobacteriaceae* in obese groups. Increased abundance of *Dialister* and reduced *Anaerostipes*, *Coprococcus*, *Lactobacilli*, *Oscillibacter*, *Atopobium* and *Alloprevotella* genus in viscerally obese groups. Increased abundance of *Parabacteroides* and *Lactobacillus* genus and reduction in *Solobacterium* and *Alloprevotella* genus in the general obese group. Positive correlation seen between sCD14 and VAT, and between Leptin and SAT area. Obese groups had higher hsCRP, leptin, sCD14 and IL-6 compared to lean groups.
8	Gelpi 2019, Denmark	Case control study of 1.PWH (n = 864) 2 HIV—(n = 75)	Years (median, IQR) 1.52 (47–60.4), 2. 59.8 (51.6–67.1)	83%	95.1% undetectable/ suppressed viral load	BMI, hip, and waist measurements; Serum measurement of hsCRP, total cholesterol, LDL, HDL and triglycerides.	PWH had higher KTR and kynurenine concentrations. QKR was associated with higher hs-CRP and neopterin concentrations while KA was associated with lower hs-CRP and neopterin concentrations. In PWH, increased waist-to-hip ratio was associated with increased KTR and QKR and decreased KA concentrations.
9	Dirajlal-Fargo 2019, USA	Randomised controlled trial of (n = 231) ART naïve PWH	Years, median 36	90%	36307.81 copies/mL (median)	Serum measurement of hsCRP, D-dimer, sCD14, sCD163, IL-6. DXA at baseline and week 96. CT measures of SAT and VAT. Insulin sensitivity was estimated by HOMA-IR.	Initially BDG significantly decreased after 4 weeks of ART initiation, but then increased at week 24 and maintained through week 96. A 2-fold higher BDG level at week 96 was associated with an 8% increase in trunk fat and a 7% increase in total fat.
10	Cheru 2018, USA	Cross sectional study of 1. PWH (on ART n = 149), 2. HIV elite controllers (n = 10) and 3. HIV negative (n = 69)	Years (mean, SD) 1. HIV 47 ± 7, 2. 53.4 ± 8, H3. IV—46 ± 7	63%	92% undeteactable viral load	Body Composition and Dietary Assessment; Serum measurements MCP-1, CXCL10, IL-6, and sCD14, HbA1c, total cholesterol, LDL, HDL, triglycerides,	I-FABP was significantly higher in PWH on ART and correlated with intake of sugar and saturated SCFA. Higher levels of serum I-FABP were inversely associated with weight, BMI, SAT, and VAT in PWH. Serum I-FABP also correlated negatively with waist and hip circumference. I-FABP correlated with MCP-1, CXCL10, sCD163, and LPS among all participants
11	Moon 2018, USA	Cross sectional study of WIHS cohort (n = 48) of PWH 1. with and 2. without DM and HIV negative 3. with and 4. without DM	Years (median, IQR) 1. 52 (51‚ 55), 2. 51 (42‚ 60), 3. 53 (52‚ 57), 4. 49 (42‚ 51)	0	1. 62.5% 2. 91.7% undetectable/ suppressed viral load 3. NA 4. NA	Targeted metabolomics of 130 metabolites: 20 amino acids, 6 biogenic amines, 12 acylcarnitines, 78 glycerophospholipids, and 14 sphingolipids	No difference in α-or β diversity by diabetes status or HIV serostatus. Four genera (*Finegoldia*, *Anaerococcus*, *Sneathia*, and *Adlercreutzia*) were less abundant in HIV+ DM+ and showed positive correlations with diabetes-associated glycerophospholipids. In HIV+ DM+, plasma levels of KTR, branched-chain amino acid and proline metabolism pathways were higher, while glycerophospholipids were lower
12	Hoel 2018, Denmark & Norway	Cross sectional study (n = 84) of PWH 1. with and 2. without DM and HIV negative 3. with and 4. without DM	Years (median, IQR) 1.54 (51‚ 58) 2.57 (54‚ 60), 3.53 (52‚ 57) 4.49 (42‚ 51)	1. 86%, 2. 90%, 3. 92%, 4. 69%	100% undetectable/ suppressed viral load	Serum glucose, HbA1c, lipid parameters. To assess endothelial dysfunction, L-arginine and ADMA were measured. Plasma levels of neopterin, kynurenine and tryptophan and the KTR was determined	The lowest α-diversity was found in PWH with DM, followed by DM alone, HIV alone, and healthy controls. MSM, Physical activity, metformin use and HDL cholesterol which were associated with higher α-diversity measures, and smoking and Framingham risk score being associated with lower α-diversity. MSM status was associated with lower predicted abundance of tryptophan metabolizing microbes. HIV+ DM+ had higher plasma KTR and higher neopterin compared to HIV- DM+ KTR and neopterin were positively correlated with ADMA and negatively correlated with L-arginine/ADMA-ratio, particularly in HIV+ DM+ patients.
13	Hove-Skovsgaard 2017, Denmark	Cross sectional study of 1. PWH with DM (n = 25), 2.PWH without DM (n = 25), 3. HIV- with DM (n = 22) and 4.HIV- without DM (n = 28)	Years (median, IQR) 1. 58 (55–61) 2. 55 (50–58), 3. 57 (55–60), 4.57 (53–60)	1. 92% 2. 96%, 3. 89%, 4. 72%	1. 26 (16–38) 2. 27 (15–40), copies/mL, (median, IQR) 3. NA 4. NA	Framingham risk score, Serum measurements of hsCRP ADMA and L-arginine	A higher ADMA and lower L-arginine/ADMA ratio was found in HIV + DM+. 50% of HIV + DM+ had hsCRP above 3 mg/L. A positive correlation between ADMA and TMAO was seen in HIV + DM+.
14	Jayanama 2022, Thailand	Cross sectional study of 1. PWH, (n = 20) with prediabetes, and 2. PWH (n = 20) with normoglycaemia	Years (mean, SD) 1.50.9¬±5.6, 2. 51.8±6.6	65%	100% undetectable/ suppressed viral load	75-g OGTT and HbA1c measurement	Lower α—diversity in the prediabetes group and a significant difference in β-diversity was observed between prediabetes and normoglycemia groups. Increased abundances of two genera in Firmicutes (*Streptococcus* and *Anaerostignum*) in the prediabetes group. Increased abundances of 13 genera (*Akkermansia*, *Gastranaerophilales*, *Desulfovibrio*, *Butyricimonas*, *Colidextribacter*, *Christensenellaceae R 7 group*, *Victivallis*, *Uncultured Bacteroidota*, *Uncultured phylum Firmicutes*, *Holdemanella*, *UCG-005*, *Eubacterium ruminantium* group, and family *Oscillospiraceae*-associated group in normoglycaemia group.

**Abbreviations**: ADMA, asymmetric dimethylarginine; ASCVD, Atherosclerotic Cardiovascular Disease, AST, aspartate aminotransferase; CT, computed tomography; DM, diabetes milletus; DXA, dual-energy X-ray absorptiometry; FLI, fatty Liver Index; FINDRISC, Finnish Diabetes Risk Scale; HDL, HbA1c, hemoglobin A1c; HDL, high density lipoprotein, HOMA-IR, homeostasis model assessment-insulin resistance; HMI, HIV-related microbiota index; I-FABP, fatty acid binding protein; KA, kynurenic acid; KTR, Kynurenine to Tryptophan Ratio; LBP, lipopolysaccharide binding protein;LDL, low-density lipoprotein; MCP-1, monocyte chemotactic protein-1; MS, metabolic syndrome; MSM, men who have sex with men; NCEP-ATP III, National Cholesterol Education Program Adult Treatment Program III; OGTT, oral glucose tolerance test; QKR, quinolinic-to-kynurenic acid ratio SAT, subcutaneous adipose tissue; SCFA, short chain fatty acids; VSURF, Variable Selection Using Random Forest; VAI, visceral Adiposity Index; VAT, Visceral adipose tissue

### Diabetes

Four studies examined diabetes and microbial diversity in PWH. Although one study found no change in diversity in PWH with diabetes (n = 48) [55], a larger cross-sectional study (n = 84) found lower α-diversity in PWH and diabetes, followed by people without HIV with diabetes [56]. Interestingly, a history of smoking and Framingham risk score were also associated with reduced α-diversity whereas physical activity, metformin use and HDL cholesterol were associated with higher α-diversity. A lower α-diversity and differences in β-diversity was also seen in a study of PWH with prediabetes compared to a normoglycaemia group [57]. In addition, two *Firmictus* genera was enriched in the prediabetes group and 13 genera were significantly higher in the normoglycaemia group. Similarly, four genera (*Finegoldia*, *Anaerococcus*, *Sneathia*, and *Adlercreutzia*) were found to be less abundant in a study of WWHV and diabetes compared to controls [55].

Although no studies specifically examined MT markers and diabetes, one investigated endothelial dysfunction in PWH with diabetes (n = 194). They found a higher asymmetric dimethylarginine (ADMA) and lower L-arginine/ADMA ratio, markers of endothelial dysfunction, in PWH with diabetes. They also found a positive correlation between ADMA and TMAO [58]. Higher ADMA levels and lower L-arginine/ADMA ratio was also found to be associated with the KTR, which was higher in a study of PWH with diabetes compared to controls [56]. Similarly, in a study of women with diabetes, irrespective of HIV status, higher plasma levels of several metabolites of the KP were found [55]. Together, these findings indicate that diabetes rather than HIV status has the biggest impact on gut microbiome changes [56].

### Obesity

Gut dysbiosis linked to obesity are well-documented in the general population but findings in PWH are mixed [59]. A small study reported lower α-diversity and elevated circulating hsCRP, IL-6, leptin and sCD14 in obese compared to lean individuals [54]. Elevated sCD14 was associated with increased visceral abdominal tissue (VAT), while higher leptin levels correlated with increased subcutaneous abdominal tissue (SAT) area. A larger study of (n = 178) PWH contradict these findings, associating higher sCD14 with lower trunk and limb fat [60]. They also found that increased LPS levels were associated with an elevated Homeostatic Model Assessment for Insulin Resistance (HOMA-IR) and triglyceride levels, together with reduced high-density lipoprotein (HDL) levels. Higher I-FABP levels correlated with sugar and saturated SCFA intake in PWH and inversely correlated with body composition, BMI, VAT and SAT [61]. Additionally, fungal translocation marker β-D-glucan (BDG) in a sub-study of a RCT of ART-naïve PWH, showed a twofold increase at week 96, correlating with an 8% rise in trunk fat and 7% increase in total fat [62]. Together, these findings suggest that although associations between inflammatory markers and metabolic risk markers occur, disparities exist with different MT markers and body composition.

Despite these variations, a more consistent link between the KP and obesity is evident in PWH. In a study of (n = 939) PWH, a 0.5-unit increase in waist-to-hip ratio was associated with 31% higher KTR, and 44% higher quinolinic-to-KA ratio with lower KA concentrations [28]. Subsequent research revealed that a higher KTR associated with elevated LBP levels, a high VAT-to-SAT ratio, and higher VAT [63]. These findings suggest dysregulated KP contributes to abdominal fat accumulation and increased gut barrier permeability, potentially fuelling inflammation in PWH.

### Metabolic syndrome

Four studies explored changes in diversity in those with MetS. One study (n = 51) found no difference in α-diversity in PWH with or without MetS [64], yet another similar-sized study (n = 60) found significantly decreased α-diversity in PWH with MetS, with a bacterial signature dominated by *Prevotella* [65]. 60% of participants in this group were MSM, possibly influencing this *Prevotella* enrichment. A larger study (n = 627) found that HIV-related microbiota were associated with increased risk of MetS and VAT accumulation, independent of sexual practice. This risk was pronounced in individuals with previous severe immunodeficiency and driven by an increase in *Desulfovibrionaceae* and decrease in *Clostridia* [8]. *Desulfovibrionaceae* produces hydrogen sulfide, harmful to the gut, while Clostridia, which are known butyrate-producers, promote gut barrier integrity [66].

Compared to MT markers, an increase in cardiovascular and inflammatory markers (PAI-1 and triglycerides-to-HDL-ratio) was seen in those with MetS compared to those without [64]. Another study found higher hsCRP together with an increase in the metabolism of several functional pathways including amino acid and energy metabolism, cofactor, vitamin and LPS biosynthesis pathways in PWH with MetS [65, 67]. Amino acid fermentation affects colonic epithelial cell energy metabolism, while LPS signalling induces inflammation and insulin resistance through MT [68]. Together, these findings suggest a proinflammatory profile in PWH with MetS.

One study employed a multi-omics approach to investigate microbial diversity, untargeted metabolomics, and their connection to diet, inflammatory and immune markers in PWH (n = 59) versus controls (n = 54) [69]. They identified 69 crucial variables (clinical, diet, immune, and microbial) and a subset of 10 highly predictive variables for MetS. Positive correlations between immune markers (LBP, ICAM-1, IL-16, IL-12, GM-CSF) and MetS were found, particulary between LBP and a high BMI. LBP binds to both microbial LPS and lipoteichoic acid, and the presence of elevated LBP in blood is indicative of increased intestinal barrier permeability [70]. Taken together, these associations suggest that inflammation originating from an impaired intestinal barrier is promoting worse metabolic health in PWH.

### Bone disease

HIV infection has been associated with low bone mineral density (BMD) and increased fracture risk [71, 72]. Emerging evidence in the general population suggests a close relationship between the gut microbiota and bone metabolism, and microbial alterations have been associated with bone health [73]. Despite this, only one eligible study compared the association between the gut microbiome and bone disease in WWHV (Table 3). They found five genera to be more abundant in those with low BMD together with a distinct metabolite profile [74], suggesting an interplay between altered gut microbiota and metabolites on BMD status in WWHV.

**Table 3:** Bone disease.

Author, year, Location	Study design	Age	% male	HIV Virological Control	NCD Examined	Main findings
Mei 2023, USA	Cross sectional study of PWH and HIV negative. Primary analysis of gut microbiota, metabolites and BMD: 58 women HIV+, 33 HIV- women. Secondary analysis of gut microbiota and plasma metabolites: 280 HIV+ and 138 HIV-	Years (mean, SD) normal BMD (n = 60), 53.2+- 4.6, low BMD (n = 31), 55.0 +- 4.5	0	1. normal BMD 81.6% and 2. low BMD 85% undetectable/ suppressed viral load	BMD measured by DXA; Untargeted metabolic profiling- A total of 467 metabolites were included	No association between α-or β- diversity and BMD status. Five genera of the *Clostridiales* order (*Dorea*, *Megasphaera*, unclassified *Lachnospiraceae*, *Ruminococcus*, and *Mitsuokella*) were more abundant in women with low BMD, and one other (unclassified *Mollicutes RF39*) was less abundant in the low-BMD group compared with the normal-BMD group. Plasma serine was higher, whereas levels of the other 16 metabolites belonging to amino acids, carnitines, caffeine, fatty acids, pyridines, and retinoids were lower in women with low BMD compared with those with normal BMD, irrespective of HIV status. *Megasphaera*, were inversely associated with several BMD-related metabolites (e.g. 4-pyridoxic acid, C4 carnitine, creatinine, and dimethylglycine).

**Abbreviations**: BMD, bone mineral density; DXA, dual-energy X-ray absorptiometry

### Liver disease

CLD has emerged as a leading cause of morbidity and mortality in PWH. Viral hepatitis coinfection and aging has been accompanied by an increased prevalence of cirrhosis and hepatocellular carcinoma (HCC) [75]. Furthermore, MAFLD has been reported in up to 35% of PWH and is expected to become the leading cause of cirrhosis in this population [76]. Eight articles relating to CLD met eligibility criteria for inclusion in this review (Table 4).

**Table 4:** Chronic liver disease.

	Author, year, Location	Study design	Age	% male	HIV Virological Control	NCD Examined	Main findings
1	Jenabian 2016, Canada	1. A cross-sectional analysis of the Canadian Coinfection Cohort of PWH (n = 14), HIV/HCV coinfection (n = 90), people without HIV and HCV (n = 12) 2. longitudinal cohort study of HIV/HCV coinfected before and after SVR (n = 34)	Years (mean, SD) PWH 46 +- 8, HIV—48 +- 8	86%	100% undetectable/ suppressed viral load	Liver fibrosis measurement using APRI score; Plasma measurement of IP-10, IL-6 and insulin.	HIV/HCV coinfection had decreased levels of Trp and increased KTR. Also had increased Kynurenine levels in fibrotic patients, that correlated with APRI scores, IP-10 and insulin levels. HIV viremia but not HCV viremia was correlated with Trp catabolism. I-FABP, LBP and sCD14 were higher in HIV/ HCV and HIV groups. Successful HCV treatment improved APRI score, markers of disease progression and MT although elevated Trp catabolism remained unchanged 6 months after SVR.
2	Balagopal 2008, USA	Cross-sectional analysis within 2 Cohort studies: The AIDS Link to Intravenous Experience [ALIVE] Cohort and the Risk Evaluation Assessment of Community Health (REACH) Cohort. PWH (n = 28),HCV (n = 88), uninfected (n = 60)	Years (mean, SD) HIV+ 35.8 ± 6.4, cirrhosis, 50.5 ¬± 6.2, non-cirrhotic 41.7 ¬± 6.0 HCV+ 4.4 ¬± 3.0	73%	Viral load not reported	Liver fibrosis	LPS, sCD14 and AAL were strongly associated with HCV-related liver disease progression (cirrhosis) AAL, a marker of liver disease in HCV-infected persons, was positively correlated with LPS and sCD14. HCV seroconversion is not significantly associated with MT.
3	Jinato 2020, Thailand	RCT of 1. HCV moninfection (n = 62), 2. HIV/ HCV coinfected (n = 24) and 3. uninfected (n = 20) pre and post treatment with an elbasvir-grazoprevir (EBR/GZR) combination	Years (mean, SD) 1. 50.3 (10.8), 2. 44.2 (7.5), 3.47.8 (9)	70%	100% undetectable/ suppressed viral load	Hepatitis C	Pretreatment α-diversity between HCV and HIV/HCV groups did not differ but was less diverse from those of healthy controls. In the HCV group, DAA therapy was associated with improved gut diversity and enrichment of *Parabacteroides* and *Subdoligranulum*) and the reduction of *Eubacterium*. In HIV/ HCV group, DAA therapy was associated with increased abundance of *Lachnospira* and *Subdoligranulum*
4	Chuaypen 2020, Thailand	Cross sectional study of people with HCV (n = 58) and HIV/HCV (n = 28)	NA	NA	Viral load not reported	Hepatitis C and measures of cirrhosis and fibrosis	No differences in diversity between groups. HIV/ HCV group had lower relative abundance of genus *Faecalibacteria* and *Blautia* Cirrhosis+ showed significant increase in *Agathobacter* and decrease in *Lachnoclostridium* when compared to cirrhosis-. *Lachnospira* was significantly higher in HIV/HCV cirrhotic patients when compared with HCV cirrhotic patients.
5	Maurice 2019, UK	Case control study of 1. PWH & liver disease (n = 33), 2. PWH without liver disease (n = 29), 3. Healthy controls (n = 17)	Years (mean, SD) 1. 46.4(12.4) 2. 48.3 (11), 3. 48 (36.5–53.5)	96%	100% undetectable/ suppressed viral load	Liver biopsy proven MAFLD graded according to the NASH CRN scoring system; Brunt criteria for liver fibrosis; Measurement of IL-6, TNF α—receptor 2, adiponectin, leptin;	sCD14 was associated with MAFLD and liver fibrosis stage MAFLD cases in PWH had higher levels of sCD14, sCD163, leptin and LAR with lower adiponectin. sCD14, sCD163 and LAR, a marker of adipose tissue dysfunction and insulin resistance increased with fibrosis stage and correlated with waist circumference.
6	Kardashian 2019, USA	Cross sectional study of the WIHS cohort of 1. PWH (n = 59), 2. HIV/HCV coinfection (n = 42), 3. uninfected controls (n = 36)	Years (media, IQR) 1. (46–55), 2. 52 (47–57), 3. 42 (37–53)	0%	1. PWH 48 (20–139), 2. HIV/HCV co-infection 80 (41–871) (median, IQR) copies/mL	VAT and SAT by MRI. HOMA-IR. Hepatic steatosis as assessed by MRS and Fibrosis calculation via FIB4	KTR was highest in the HIV/HCV-group, followed by PWH, then the uninfected group. PWH and HIV/HCV coinfection had higher FIB-4 scores than the uninfected group. A greater kynurenine level (per doubling), was associated with 29.6% greater FIB-4. Higher KTR was significantly associated with LSM. HIV/HCV coinfection was associated with a 94% higher LSM.
7	Martinez-sands 2023	Cross sectional study of 1. 30 HIV+MASLD+, 2. 30 HIV+MASLD- and 3. 20 HIV-MASLD+	Years (median, IQR) 1. 54 (43,59) 2. 53 (45, 56) 3. 56 (51, 68)	80%	Viral load not reported	Presence of transaminitis Liver Fibroscan	Higher α-diversity in those with MAFLD Distinct beta diversity in MAFLD in both PWH and HIV negative The most abundant genera in MAFLD- were *Prevotella*, *Bacteroides*, *Dialister*, *Acidaminococcis*, *Alloprevotella* and *Catenibacterium*; in MAFLD+, the most enriched genera were *Ruminococcis*, *Streptococcus*, *Holdemanella*, *Blautia and Lactobacillus*.
8.	Martin 2023	Cross sectional study of the MASH Cohort of PWH who use cocaine or no drugs at all 1.36 PWH with **FIB-4 < 1.45** **2. 14 PWH with FIB-4 ≥ 1.45**	Years (mean, SD) 1.54.5 ± 7.5 2. 56.2 ± 4.6	58%	68% undetectable/ suppressed viral load	24 hour dietary recall Fibrosis calculation via FIB4 and liver enzyme measurements	Healthy eating index score was lower than the national average. Compared to participants with FIB-4 ≥ 1.45, participants with FIB-4 < 1.45 had significantly higher intake of dairy The RA of *Ruminococcaceae*, *Roseburia* species, and *Lachnospiraceae* were higher in participants with FIB-4 < 1.45 The RA of the *Bacteroidales* and *Cardiobacteriales* orders, *Bacteroidia* class, *Bacteroidetes* phylum, *Cardiobacteriaceae* family, and *Cardiobacterium* genus were higher in those with FIB-4 ≥ 1.45

**Abbreviations**: AAL, Aleuria aurantia lectin; APRI, aspartate aminotransaminase to platelet ratio; CRN, Clinical Research Network; hsCRP, high‐sensitivity C‐reactive protein; DAA, direct acting antivirals; FIB-4, fibrosis 4,HCV; hepatitis C virus; IL-6, interleukin‐6; KTR, Kynurenine to Tryptophan Ratio; LAR, Leptin to adiponectin ratio; LPS, lipopolysaccharide; LSM, Liver Stiffness Measurement; MRS, magnetic resonance spectroscopy; MT, microbial translocation, RCT, Randomised controlled trial; SAT, subcutaneous adipose tissue; sCD14, soluble CD14; Trp, Tryptophan; VAT, Visceral adipose tissue

Two studies explored the impact of HCV infection on bacterial composition in PWH. One study found no differences in bacterial diversity between HCV and HIV/HCV coinfected groups [77] nor were there differences in HCV or HIV/HCV coinfected groups in another study pre and post direct-acting antiviral (DAA) treatment for HCV [78]. Both studies did describe differences in bacterial composition between groups with improvements in gut diversity following DAA therapy, including enrichment of some beneficial bacteria and reductions of pathogenic bacteria. Together these studies suggest that co-infection with HCV does not influence overall bacterial diversity in PWH, but that a microbiota signature may exist in co-infected PWH.

In studies of MAFLD, one study found distinct beta-diversity and microbial composition in those with MAFLD, regardless of HIV status [79]. In contrast, a similar-sized study found no difference in the bacterial composition between PWH with and without MAFLD or fibrosis [80]. These studies differed in their definition of MAFLD however, with the former study screening participants based on the presence of transaminitis and the latter study including participants with liver-biopsy proven MAFLD. A recent study of the Miami Adult Studies on HIV (MASH) cohort explored the association between diet and liver fibrosis in PWH [81]. They found that a lower dairy intake was seen in participants with a higher FIB-4 score, yet the study also found that participants in this study displayed poorer diet quality compared to the general U.S. population. They also found differences in bacterial composition based on FIB-4 score, with a higher relative abundances of butyrate-producing taxa seen in those with a low FIB-4 score. These studies suggest that the composition of gut microbiota may play a role in MAFLD, with potential implications for dietary interventions however further studies are warranted to understand these associations in PWH with MAFLD.

In addition to composition changes, three studies included associations between MT markers and CLD. Following HCV infection, both MT markers (I-FABP, LBP, sCD14) and kynurenine levels were found to be higher in coinfected and monoinfected individuals compared to uninfected controls [82]. Following HCV treatment, these markers all decreased suggesting that inflammation associated with HCV viraemia may be a driver of these MT responses. In other studies of CLD, LPS, sCD14 and AAL were strongly associated with development of cirrhosis among those with HIV/HCV coinfection, and sCD14 was found to be correlated with the presence of MAFLD and liver fibrosis stage [80, 83]. Overall, this suggests that these markers may serve as indicators of CLD severity or progression in HIV/HCV coinfection.

Two studies of the KP both found that the KTR was highest in HIV/HCV coinfection and one found that a doubling of kynurenine levels was associated with 29.6% increase in FIB-4 scores [82, 84]. In HCV/HIV subjects, kynurenine was primarily elevated in fibrotic patients and correlated with IP-10, a marker of HIV and HCV disease progression, as well as aspartate aminotransaminase to platelet ratio (APRI scores) and insulin levels. Kynurenine levels and the KTR showed no change before and after HCV treatment, but there was an improvement in the APRI score. Both these studies suggest that KTR may modify the association between HIV infection and higher fibrosis in HCV co-infection.

### Cancer

As PWH age, the risk of cancer increases. Most HIV-associated cancers are caused by oncoviruses such as HHV-8, Epstein–Barr virus (EBV), high-risk human papillomavirus (HPV), HBV, HCV, and Merkel-cell polyomavirus [85]. HIV-associated and HIV non-associated cancers were included in our search terms for this review, and 3 studies met eligibility criteria (Table 5).

**Table 5:** Cancer.

	Author, year, Location	Study design	Age	% male	HIV Virological Control	NCD Examined	Main findings
1	Serrano-Villar 2017, Spain	Cross sectional (n = 42) study of MSM with HIV and MSM Without HIV Infection	Years (mean, SD) 39 ¬± 9 years	100	95% undetectable. Suppressed viral load	Anal screening for HPV lesions; study participants were classified according to the presence of histologic lesions: HSIL (N = 14), LSIL (N = 13), and normal (N = 15)	α-and β- diversity was higher in rectal mucosa compared with faecal samples with distinct microbiota compositions in both sample type. *Prevotella* was the most abundant genus in both mucosa and faeces. In mucosa samples, *Peptostrepcoccus* and *Anerovibrio* were enriched in those with AIN, *Campylobacter* was enriched in those with LSIL and *Gardnerella* & *Catenibacterium* were enriched in those with HSIL. In faecal samples, those with AIN showed depletion of *Bifidobacterium*, and enrichment for *Peptostreptococcus*. *Campylobacter* was enriched in those with LSIL, whereas *Ruminococcus* and *Pseudomonas* were predictive of HSIL.
2	Merlini 2018, Italy	Cross sectional study of PWH (n = 36)		100	100% undetectable/ suppressed viral load	Anal HPV genotyping by qPCR and sequencing analysis.	aHPV patients showed a marked dysbiosis, with higher proportion of *Prevotellaceae* and lower *Leuconostocaceae* Participants with hrHPV cytology displayed a higher proportion of *Prevotellaceae* and *Veillonellaceae*, but lower *Bacteroidaceae*, *Lachnospiraceae* and *Rikenellaceae* as compared to lrHPV cytology. Predicted metabolic function showed that PWH with hrHPV genotypes showed increased abundance of genes related to immune system activation and to metabolic syndrome
3	Marks 2013, USA	Case control study of PWH (n = 246) from 4 cohort studies: The DCG; The MHCS I and MHCS II; and the ACC classified as 1. NHL cases (n = 56) and 2. controls (n = 190)	Age by ranges (years), Cases- NHL <30–15 (26.8%) 30-36- 11(19.6%) 37-42- 17 (30/4%) Controls <30–37 (19.5%) 30-36- 51 (26.8% 37–42 52 (27.4%) >43–50 (26.3%)	1.94.6% 2.93.7%-	26.5% viral load < 91,201 copies per ml, 20% viral load >91,201 copies per ml, 57% NA	NHL	sCD14 and LPS were associated with a 2.5-3-fold increased NHL risk in PWH. Among cases, levels of sCD14 were positively correlated with levels of lambda free light chains.

**Abbreviations**: ACC, AIDS Cancer Cohort Study; AIN, Anal intraepithelial neoplasia; aHPV, altered HPV; DCG, The District of Columbia/New York gay men’s cohort; HPV, Human papillomavirus; hrHPV, high risk HPV; lrHPV, low risk HPV; LPS, lipopolysaccharide; MHCS, The Multicenter Hemophilia Cohort Studies; MSM, men who have sex with men; NHL, non-hodgkin lymphoma; sCD14, soluble CD14

In a study of HPV-associated anal cytology changes (n = 36), a marked gut dysbiosis was evident in PWH with altered anal HPV and a distinct composition difference was seen in those with high-risk HPV compared to low-risk HPV genotypes [86]. Conversely, no differences in the MT markers sCD14, LPS, calprotectin and I-FABP were seen between these groups. Another study found distinct diversity changes in samples collected from rectal mucosa biopsies compared to faecal samples and suggest that certain prevalent taxa taken from rectal biopsies could be used as diagnostic markers for particular histological- graded lesions [87]. Finally, a large retrospective cohort study (n = 246) of lymphoma cases in the US [88] found elevated circulating levels of sCD14 and LPS were associated with a 2.5–3 fold increased risk of non-Hodgkins lymphoma (NHL) in PWH, with sCD14 positively correlated with levels of lambda free light chains. These studies suggest that the presence of certain proinflammatory or pathogenic bacteria are associated with HPV-associated pre-cancerous changes and that although MT markers may not be associated with HPV, they could be prognostic in certain cases of NHL.

### Frailty

Impairments in physical function among ART-treated PWH have been linked to systemic inflammation. Emerging evidence supports an association between the gut microbiome and physical function/frailty [89]. Three studies on frailty met our eligibility criteria (Table 6). A small study (n = 36) found no significant differences in overall bacterial diversity, physical function, and body composition between older PWH and controls [90]. However, the gut microbiome composition in older PWH differed from that in older negative controls, suggesting a unique aging gut profile in PWH. They also found that higher faecal levels of the SCFA butyrate was associated with increased grip strength in both groups. In contrast, a second similar sized study (n = 36) did find diversity differences between older PWH and HIV negative controls, but found no changes between frail or non-frail groups controls as measured by the Fried Frailty Index [91]. The larger HIV Neurobehavioral Research Center study (n = 398) identified a distinct gut microbiome in PWH with impairment in activities of daily living (ADLs) that was enriched in *Bacteroides* [92], which has previously been associated with cognitive decline in other studies of PWH [93]. Despite different frailty assessments, all studies imply unique microbiome compositions in older, frailer PWH, emphasising the complexity of studying and measuring frailty in aging.

**Table 6:** Frailty.

Author, year, Location	Study design	Age	% male	HIV Virological Control	NCD Examined	Main findings
Taylor 2020, USA	Cross sectional study of the HNRC study of 1. PWH (n = 200), 2. HIV/HCV coinfection (n-46) 3. Uninfected controls (n = 101) Participants categorised as (a) dependent in IADLs or (b) independent	Years (mean, SD) 1.(a) 54.7 (11.1), (b)- 50.0 (12.2), 2. (a) 54.7 (10.6), (b) 53.0 (8.1) 3.(a) 48.8 (18.4), (b)- 51.3 (16.4)	1. (a) 86%, (b) 87%; 2. (a) 77%, (b); 92% 3.(a) 60%, (b) 60%	1. 87% 2. 88% 3. 72% 4. 94% undetectable/ suppressed viral load	Depression, diagnosed by DSM-IV, Beck Depression Inventory-II Assessments of ADLs with a modified version of the Lawton and Brody Scale.	Differences in β- diversity seen between independent and dependent PWH and HIV/ HCV group and displayed distinct microbiome compositions. Those who reported reduced independence were enriched in genus *Bacteroides* in PWH and HIV/ HCV groups. IADL dependence was significantly associated with greater neurocognitive impairment, unemployment, disability, depressed current mood, and higher rates of DSM-IV diagnoses of lifetime and current MDD, and methamphetamine use disorder.
Dillon 2021, USA	RCT post-hoc analysis of the ’Exercise in Healthy Aging’ study and the ’Assessing Tenofovir Pharmacology in Older HIV-infected Individuals Receiving Tenofovir-based Antiretroviral Therapy’ study of 1.PWH (n = 22) and 2.Uninfected controls (n = 22)	Years (mean, SD) 1. 60.79 (7.71) 2. 59.50 (6.62)	95%	100% undetectable/ suppressed viral load	Functional assessments include: Time to complete a 400m walk, climb a flight of 10 stairs, or to complete 10 repeat chair rises, SPPB test, hand grip strength assessment, muscle strength measurements. Muscle mass estimate of LBM, and ALM DXA	No difference between α-or β- diversity seen between groups. Lower relative abundance of *Lachnospiraceae* and *Alistipes* in PWH versus controls. Greater abundance of *Alistipes*, *Escherichia*, *Prevotella*, *Megasphaera*, and *Subdoligranulum* associated with reduced lower extremity muscle function, decreased lean mass, or lower SPPB scores. Greater abundance of *Dorea*, *Coprococcus*, and *Phascolarctobacterium* in older PWH were associated with better muscle function, lean mass, and SPPB scores. In both groups, increasing levels of butyrate were significantly associated with greater grip strength
3.	Sanchez-Conde 2023	Cross sectional study of 1. 24 PWH and 2. 12 HIV negative controls	Years (mean, SD) 1. 61.9 (7.6) 2.60.6 (6.1)	100% undetectable/ suppressed viral load	77&	Frailty according to Fried’s frailty phenotype, defined by 5 functional criteria: shrinking, weakness, poor endurance and energy, slowness, and low physical activity level

**Abbreviations**: ADL, activities of daily living; ALM, appendicular lean mass; DSM-IV, The Diagnostic and Statistical Manual of Mental Disorders, Fourth Edition; DXA, dual-energy X-ray absorptiometry; HCV; hepatitis C virus; HNRC, HIV Neurobehavioral Research Centre; IADLs, Instrumental activities of daily living; LBM, lean body mass; MDD, major depressive disorder; SPPB, Short Physical Performance Battery

### Neuro-psychiatric conditions

The term “gut-brain axis” is a bidirectional communication network that links the enteric and central nervous systems (CNS). Explored in conditions like Alzheimer’s, Parkinson’s [94], and Multiple Sclerosis [95], it extends also to neurocognitive and mood disorders [96]. Seven articles relating to mental health and neurological disorders met eligibility criteria (Table 7).

**Table 7:** Mental Health and neurological disorders.

	Author, year, Location	Study design	Age	% male	HIV Virological Control	NCD Examined	Main findings
1	Lyons 2011, USA	Cross sectional study of PWH with nadir CD4 count <300 (n = 97)	Years (mean, SD) 47.1 ± 8.0	76%	14,924 (undetectable/ suppressed, 843,720) (median, IQR) copies/mL	HAND diagnoses were determined using AAN diagnostic criteria Plasma and CSF HIV RNA	Elevated plasma sCD14 seen with GCI, impaired testing in attention and learning domains compared to unimpaired subjects. Plasma sCD14, Plasma and CSF VL, current and nadir CD4 count, and plasma CCL2 and LPS levels did not differ by HAND diagnosis
2	Zhang 2019, China	Cross sectional study of PWH (n = 85)	Years (median, IQR) 33 (27–41)	HAND 89.7%, non-HAND 87.8%	39,810.72 (6,309.57, 100,000) (median, IQR) copies/mL	HAND diagnosis by MoCA cut-off 26/30	The HAND group had lower α-diversity than the non-HAND group, but this did not remain when matched for confounders. No significant differences in the β- diversity or predictive function of the gut microbiota was found between the HAND and non-HAND groups.
3	Ellis, 2022, USA	Case control study of 1.PWH (n = 226) and 2.HIV negative (n = 101)	Years (mean SD) 1. 52.4 ¬± 11.8, 2. 51 ¬± 16.7	87.60%	90% undetectable/ suppressed viral load	DSP diagnosed by clinical exam. Self-reporting tool and clinical examination for DNP diagnosis.	Severe DNP was associated with lower α-diversity but not changes in β- diversity. Found relative increases in the ratios of *Blautia* and *Clostridium* to *Lachnospira* in PWH with DNP. Rates of DSP and DNP were not different in MSM and non-MSM PWH, however, MSM PWH had less severe DNP than non-MSM.
4	Perez-Santiago 2017, USA	Cross sectional study of 1. PWH (n = 96) and 2. HIV negative (n = 30)	Years (median, IQR) 1. 55 (49.5–61.3), 2. 58 (48.8–69)	88%	Not reported	HAND, diagnosed by neuropsychological assessment and self-reported depression screening.	PWH with HAD had significantly lower levels of *Firmicutes* and higher levels of *Bacteroidetes* Higher levels of *F*. *prausnitzii* is associated with higher depression and higher levels of *B*. *caccae* are associated with better cognition
5	Taylor 2020, USA	Cross sectional study of 1. PWH (n = 219), 2. HIV/ HCV co-infection (n = 48) and 3. uninfected controls (n = 106)	Years (mean, SD) 1. 51.7 (12.0), 2. 53.8 (9.1), 3. 51.2 (16.3)	1. 88%, 2. 85%, 3. 60%	93% undetectable/ suppressed viral load	DSM-IV diagnosis of lifetime major depressive disorder was evaluated using CIDI and BDI-II.	The HIV/HCV group had a significant difference in bacterial composition between those who met lifetime diagnostic criteria for MDD versus those who did not and were enriched in primary and secondary bile acids. Depression-related taxa found in HIV/HCV group include *Enterobacteriaceae*, *Alistepes onderdonkii*), *Bacteroides* and *Parabacteroides distasonis*.
6	Dong 2021, China	1. PWH with NCI (n = 67) and 2. without NCI (n = 35) from the Comparative HIV and Aging Research in Taizhou (CHART) cohort study	Years (mean, SD) 1. 54.6 +- 9.7, 2. 55.3 +- 9.3	82.10%	Not reported	Neurocognitive tests for global cognitive score. Zung Self-Rating Depression Scale. Jenkins four-item sleep questionnaire for insomnia. Fried Frailty criteria Vitamin D Measurements CIMT measurement by US	No difference in α-or β- diversity between the NCI and non-NCI groups. The NCI group had higher CIMT, carotid plaque, cholesterol and increased abundance of *Spirochaetes* and *Epsilonbacteraeota* than non-NCI. 14 metabolites [bile acids (BAs), glycerophosphoinositols, fatty acids, eicosanoids, and fatty amides] were significantly increased in the NCI group. There were negatively correlated with *Faecalibacterium*, *Corprococcus*_2, and *Ruminococcus*_1 and positively correlated with *Klebsiella* Four vitamin D metabolites, five terpenoids and resolvin D1 were significantly decreased in the NCI group. These were positively correlated with *Faecalibacterium*, *Corprococcus*_2, and *Ruminococcus*_1, and negatively correlated with *Klebsiella*. Vitamin D level was negatively associated with CIMT and with *Ruminococcus*_1, *Eubacterium*_*eligens*_group, and *uncultured*_*bacterium*
7.	Hua, 2023	1. PWH (n = 302) and 2. HIV negative (n = 144)) from the WIHS Cohort.	Years (mean, SD) 53.1	0%	74.2% undetectable/ suppressed viral load	A neuropsychological test battery assessing the following domains: learning, memory, psychomotor speed, attention/working, motor function, verbal fluency and executive function	α-diversity was higher in women with NCI compared to those without. No differences in beta diversity. Found a higher abundance of *Methanobrevibacter*, *Odoribacter*, *Pyramidobacter*, *Eubacterium*, *Ruminococcus*, and *Gemmiger*, and lower abundance of *Veillonella* were associated with NCI, this was more profound in PWH compared to HIV− women. Most associations with bacterial taxa were observed for learning and memory.

**Abbreviations**: AAN, American Academy of Neurology; BDI-II, Beck Depression Inventory-II; CIDI, Composite International Diagnostic Interview; CIMT, Carotid intima media thickness; CSF, cerebrospinal fluid; DNP, distal neuropathic pain; DSP, distal sensory polyneuropathy; GCI, global cognitive impairment; HAD, HIV-associated Dementia; NCI, neurocognitive impairment; HAND, HIV-associated neurocognitive disorder; LPS, lipopolysaccharide; MoCA, Montreal Cognitive Assessment; MSM, men who have sex with men; sCD14, soluble CD14; US, ultrasound; VL, viral load

A small cross-sectional study of ART-naïve PWH (n = 85) did not find associations between gut microbial dysbiosis and HIV-associated neurocognitive disorders (HAND). Despite a relatively young cohort (median age 33), 46% met HAND criteria by the Montreal Cognitive Assessment (MoCA) test [97]. A similar sized study (n = 102) found no diversity differences between PWH with and without neurocognitive impairment (NCI) but noted varied microbial abundance, including increased *Spirochaetes* and *Epsilonbacteraeota* in the NCI group [98] and decreased abundance of butyrate-producing bacteria in the non-NCI group. In contrast, a larger cross-sectional study of women with and without HIV (n = 466) did find higher α-diversity measures in women with NCI compared to those without. They also found a distinct microbial composition associated with NCI and this was more pronounced in WWHV [99]. These studies suggest that while gut microbial diversity might not directly correlate with HAND in certain populations, specific microbial abundances may be associated with NCI in PWH, with variations observed by gender.

Other studies have explored gut dysbiosis associated with other neurological conditions. In measures of depression, PWH (n = 96) had increased abundance of *F*. *prausnitzii* [100] and PWH co-infected with HCV with a history of Major Depressive Disorder (MDD) had enrichment of *Enterobacteriaceae*, *Alistepes onderdonkii*, *Bacteroides* and *Parabacteroides distasonis* [101]. In measures of distal neuropathic pain (DNP), neuropathic parasthesia and neuropathic sensory loss, a lower α-diversity was seen in PWH together with an increased ratio of *Blautia* and *Clostridium* to *Lachnospira* [102] (Table 7).

In addition to compositional changes, the MT marker sCD14 was found to be elevated in subjects with NCI in a cohort of PWH (n = 97) with nadir CD4 counts <300 cells/mm^3^ [103]. Elevated sCD14 was seen in subjects with impaired testing in attention and learning domains and correlated inversely with global, attention, and learning T scores, suggesting these domains are the main drivers of impairment [98]. In addition to MT changes, the NCI group had higher CIMT, CAP and cholesterol than the non-NCI group. Together, these studies suggest that overall microbiome diversity in HAND is apparently unaltered, but that a gut microbiota composition signature may exist in certain neurocognitive conditions including HAND, DNP and MDD in PWH. Future studies in neuro-psychiatric conditions should also explore the role of other MT markers and metabolites, including those of the KP.

## Discussion

This systematic review is the first to summarise the evidence of age-related comorbidities and their associations with gut dysbiosis in chronically-infected PWH. We describe studies that examine alterations in gut diversity, composition, MT and microbiota-derived metabolites, revealing overlapping and interacting contributions to NCD development (Fig 2).

Across studies, gut diversity was consistently lower in PWH compared to controls, but diversity changes differed depending on the NCD assessed. For instance, no difference in diversity was seen in HAND vs non-HAND groups [97, 98, 100] yet significant changes were seen in those with DNP [102] and a lifetime history of depression [101]. Examining HAND classification in these studies reveals significant variation in diagnostic criteria with the lack of a consensus on HAND definition complicating research in this field [104]. Similarly, conflicting results of diversity changes are seen in studies of frailty [90–92], MAFLD [79, 80] and CVD [36, 40, 105].

In addition to diversity, microbial composition differed between PWH and controls and also by NCD. In studies of frailty, bacterial compositions were distinct between frail versus non-frail groups and between PWH and controls, suggesting that a unique gut-physical function axis exists in PWH [90, 92]. Similarly, in studies of HPV-related cancer, compositions differed depending on both histological grade [86] and the type of sample studied (rectal mucosa versus faecal samples) [87]. Distinct compositional differences are also found between body type (lean, generally obese and viscerally obese) [54] and between those with prediabetes and normoglycaemia [57]. In contrast, assessments of diversity were conflicting in studies of HIV/HCV coinfection. However, these studies differed in their design and in the outcomes being assessed. For instance, some studies focused on the impacts of chronic HCV infection on the gut microbiome [77] whilst others compared gut dysbiosis pre- and post-DAA treatment [78]. Overall, these studies show that compositional changes and not diversity may be more important in differentiating risk of NCD in PWH.

The most commonly assessed MT markers were sCD14 and LPS and they have been associated with progression of atherosclerosis [41], hypertension [43] and with HCV-related liver disease progression [83]. sCD14 has been linked with HAND diagnosis [103], increased risk for NHL [88], liver fibrosis and MAFLD [80] in PWH. Higher plasma sCD14 levels have previously been associated with immune activation and all-cause mortality in PWH [106, 107] and so it comes as no surprise that this marker is also associated with progression of NCD in HIV. However, sCD14 serves as a biomarker of monocyte activation and reflects LPS-induced monocyte activation but is not a specific MT marker. Furthermore, the relationship between sCD14 and LPS is complex in PWH, with increased sCD14 associated with high LPS [108], low LPS levels [109] and neither [107] in different studies. This variability may be due to laboratory difficulties, HIV disease severity, ART duration [110], genetic factors [111] and the intestinal microbiota [112]. In addition, LPS may respond to stimuli other than endotoxins from gut bacteria [113], complicating it’s interpretability.

Mixed findings in linking MT markers with MetsS may stem from variations in methods used to assess MetS [8, 60, 64]. Some studies measured levels of lipoproteins and other atherosclerotic markers, whilst others used DEXA scan or CT scan measurements of body composition. Furthermore, only half of MetS studies included uninfected controls, complicating the distinction between HIV-related changes and metabolic changes. Other MT markers of interest include the fungal marker BDG and I-FABP, an indicator of enterocyte damage. Studies found that these markers associated with increased adiposity in PWH, suggesting that intestinal damage is linked with nutrient malabsorption and inflammation [61, 62]. Further investigation of these markers with NCDs could offer insights into gut barrier disruption and systemic inflammation.

The gut microbiota produces SCFA and various metabolites, such as those of the KP and choline metabolism, involved in multiple human physiological pathways. The KTR is consistently higher in PWH compared to controls [28, 56, 82] and KP metabolites are associated with vascular endothelial dysfunction [52], CAP development [39], liver fibrosis [84] and increased adiposity [28] in PWH. Choline metabolites (TMA and TMAO) are similarly associated with progression of atherosclerosis [114], CAP [48] and also myocardial fibrosis [51]. Whilst these studies draw conclusions based on associations, a further understanding of the mechanistic links driving these associations is needed in order to draw meaningful conclusions.

All studies included in this review were conducted in people with chronic HIV, predominantly with suppressed viral loads. Only two studies compared treated and untreated HIV directly [60, 115]. One study found that TMAO was linked to sCD14 in untreated people with HIV, but not to platelet hyperreactivity in either group. The other found higher triglycerides and insulin resistance in untreated people, and an association between MT and lower HDL in treated HIV, with no body composition differences between groups. These studies suggest some possible differences in NCD measures between treated and untreated chronic HIV infection, but few studies exist due to early ART initiation.

The strengths of this review include: (1) a registered review protocol with a comprehensive search strategy across five databases; (2) evaluation of many studies that include an older population; (3) assessment of populations chronically infected with HIV and (4) evaluation of microbiome composition, MT markers and gut microbiota-derived metabolites as tools for assessing the gut microbiome’s impact on comorbidities in PWH. However, limitations include few studies involving gut biopsies, technical variations with MT assays and responses, cross-sectional study designs preventing causal inferences and a lack of diversity in terms of geography, ethnicity, socioeconomic status and gender. Finally, whilst renal disease is commonly reported in PWH, notably no studies looked specifically at associations with this comorbidity.

## Conclusions

The burden of age-related comorbidities causes significant morbidity and mortality for PWH. The lack of prospective studies that link changes in gut dysbiosis to NCD development remains a significant research gap. Further studies on the mechanistic links between gut dysfunction, inflammation and these comorbidities is vital for developing new diagnostic and therapeutic approaches in PWH.

## Supporting information

S1 Table PRISMA 2020 checklist. (DOCX)

S2 Table Table summary of search strategy and searching strings for the Pubmed, Scopus and Embase databases. (DOCX)

S3 Table Quality appraisal result of included studies; using Joanna Briggs Institute (JBI) quality appraisal checklist for cross-sectional study designs. (DOCX)

S4 Table Quality appraisal result of included studies; using Joanna Briggs Institute (JBI) quality appraisal checklist for cohort study designs. (DOCX)

S5 Table Quality appraisal result of included studies; using Joanna Briggs Institute (JBI) quality appraisal checklist for case-control study designs. (DOCX)

S6 Table Quality appraisal result of included studies; using Joanna Briggs Institute (JBI) quality appraisal checklist for randomised control study designs. (DOCX)

S7 Table All data extracted from the Pubmed, Embase and Scopus databases. (XLSX)

S8 Table Quality appraisal result of included studies; using Joanna Briggs Institute (JBI) quality appraisal checklist for each study type. (XLSX)

S9 Table All studies identified in the literature search, including those that were excluded from the analyses. (XLSX)
