# Supplementary material for: HIV, the gut microbiome and clinical outcomes, a systematic review
Source: PLoS One. 2024 Dec 9;19(12):e0308859. doi: 10.1371/journal.pone.0308859 (PMC11627425; doi:10.1371/journal.pone.0308859)
Supplement: S2 Table — (DOCX) [file pone.0308859.s002.docx]

# **Supplementary Data**

**S2 Table**: **Search strategy and searching strings for the Pubmed, Scopus and Embase databases.**

| 1 | (“HIV” OR “Human Immunodeficiency Virus” OR "human immuno-deficiency virus" OR “HIV/AIDs” OR “AIDS virus”) | ti,ab, kw |
| --- | --- | --- |
| 2 | (“gut microbiome” OR “gastrointestinal microbiome” OR “gastrointestinal microbiota” OR “gut microbiota” OR “gut microflora” OR “gastrointestinal flora” OR “intestinal microbiome” OR “intestinal microbiota”) | ti, ab, kw |
| 3 | #1 AND #2 |  |
| 4 | (“inflammation” OR “immune activation”) | ti, ab, kw |
| 5 | (“comorbidities” OR “multimorbidity”) | ti, ab, kw |
| 6 | (“chronic disease” OR “age-related disease” OR “chronic illness” OR “chronic condition” | ti, ab, kw |
| 7 | (“frailty” OR “frailness” OR “frailty syndrome” OR “debility” OR “debilities”) | ti, ab, kw |
| 8 | (“cardiovascular disease” OR “heart disease” OR “coronary disease” or “myocardial infarction” OR “hypertension” OR “stroke” OR “embolic event” OR “cerebrovascular accident” OR “brain ischaemia” OR “intracranial haemorrhage”) | ti, ab, kw |
| 9 | (“metabolic disease” OR “diabetes” OR “diabetes mellitus” OR “diabetes type 2” OR “non-insulin dependent diabetes”) | ti, ab, kw |
| 10. | (“dyslipidemia” OR “hypercholesterolemia” OR “high cholesterol” ) | ti, ab, kw |
| 11. | (“Obesity” OR “overweight” OR “abdominal obesity” OR “morbid obesity” OR “metabolically benign obesity”) | ti, ab, kw |
| 12. | (“bone disease” OR “osteoporosis” OR “osteopenia” OR “bone density” OR “fractures, bone”) | ti, ab, kw |
| 13. | (“renal disease" OR “kidney disease” OR “chronic kidney disease” OR “Kidney failure” OR “kidney dysfunction” OR “renal insufficiency” OR “kidney insufficiency”) | ti, ab, kw |
| 14. | (“hepatitis” OR “liver disease” OR “liver failure” OR “cirrhosis” OR “MAFLD” OR “nonalcoholic Steatohepatitis” OR “AFLD” OR “hepatocellular carcinoma” OR “liver cancer”) | ti, ab, kw |
| 15. | (“neurocognitive disease” OR “cognitive impairment” OR “dementia” OR “HIV Associated Neurocognitive Disorders” OR “HAND” OR “neurocognitive disorders” OR “cognitive disorders”) | ti, ab, kw |
| 16. | (“malignancy” OR “sarcoma” OR “neoplasm” OR “oncology” OR “malignant” OR “carcinoma” OR “lymphoma” OR “melanoma” OR “tumor” OR “tumour” OR “leukemia” OR “leukaemia”) | ti, ab, kw |
| 17. | #4 OR#5 OR #6 OR #7 OR #8 OR #9 OR #10 OR #11 OR #12 OR #13 OR #14 OR #15 OR #16 |  |
| 18. | # AND #17 |  |
| 19. | limit #18 to yr="2012 -Current" |  |
| 20. | Limits #19 to “human studies” |  |
| 21. | “Randomized control trial” OR “clinical trial” |  |
| 22. | “Cohort study” OR “follow up studies” OR “longitudinal studies” OR “prospective studies” OR “retrospective studies” |  |
| 23. | “Case-control study” OR “retrospective studies” |  |
| 24. | “Cross-sectional study” |  |
| 25. | #20 and #21 OR #22 OR #23 OR #24 |  |
