# Supplementary material for: HIV, the gut microbiome and clinical outcomes, a systematic review
Source: PLoS One. 2024 Dec 9;19(12):e0308859. doi: 10.1371/journal.pone.0308859 (PMC11627425; doi:10.1371/journal.pone.0308859)
Supplement: S3 Table — (DOCX) [file pone.0308859.s003.docx]

**S3 Table. Quality appraisal result of included studies; Using Joanna Briggs Institute (JBI) quality appraisal checklist for Cross-Sectional Study designs**

| **Authors** | **D1** | **D2** | **D3** | **D4** | **D5** | **D6** | **D7** | **D8** | **Overall Score** |
| --- | --- | --- | --- | --- | --- | --- | --- | --- | --- |
| Amador-Lara 2022 | Yes | Yes | Yes | Yes | Yes | Yes | Yes | Yes | 8 |
| Armstrong 2021 | Yes | Unclear | Yes | Yes | Yes | Yes | Yes | Yes | 7 |
| Blodget 2012 | Yes | Yes | Yes | Yes | Yes | Yes | Yes | Yes | 8 |
| Cheru 2018 | Yes | Yes | Yes | Yes | Yes | Yes | Yes | Yes | 8 |
| Chuaypen 2020 | Unclear | No | Yes | Yes | Unclear | Unclear | Yes | Yes | 4 |
| Colaco 2021 | Yes | Yes | Yes | Yes | Yes | Yes | Yes | Yes | 8 |
| Dillon 2021 | Yes | Yes | Yes | Yes | No | No | Yes | Yes | 6 |
| El-Far 2021 | Yes | Yes | Yes | Yes | Yes | Yes | Yes | Yes | 8 |
| Gelpi 2022 | Yes | Yes | Yes | Yes | Yes | Yes | Yes | Yes | 8 |
| Gogokhia 2020 | Yes | Yes | Yes | Yes | No | No | Yes | Yes | 6 |
| Haissman 2016 | Yes | Yes | Yes | Yes | Yes | No | Yes | Yes | 7 |
| Haissman 2017 | No | Yes | Yes | Yes | Yes | No | Yes | Yes | 6 |
| Hoel 2018 | Unclear | Unclear | Yes | Yes | Yes | Yes | Yes | Yes | 6 |
| Hove-Skovsgaard 2017 | Yes | Yes | Yes | Yes | Yes | Yes | Yes | Yes | 8 |
| Jayanama 2022 | Yes | Yes | Yes | Yes | Yes | No | Yes | Yes | 7 |
| Jenabian 2016 | Yes | Yes | Yes | Yes | Yes | No | Yes | Unclear | 6 |
| Kardashian 2019 | Yes | Yes | Yes | Yes | Yes | Yes | Yes | Yes | 8 |
| Kehrmann 2019 | Yes | Yes | Yes | Yes | Yes | Unclear | Yes | Yes | 7 |
| Knudsen 2016 | No | No | Yes | Yes | Yes | No | Yes | Yes | 5 |
| Lyons 2011 | Yes | Yes | Yes | Yes | Yes | Yes | Yes | Yes | 8 |
| Mei 2023 | Yes | Yes | Yes | Yes | Yes | Yes | Yes | Yes | 8 |
| Merlini 2018 | Yes | No | Yes | Yes | No | No | Yes | Unclear | 4 |
| Montrucchio 2020 | Yes | Yes | Yes | Yes | Yes | Yes | Yes | Unclear | 7 |
| Moon 2018 | Yes | Yes | Yes | Yes | Yes | Yes | Yes | Yes | 8 |
| Perez-Santiago 2017 | No | No | Unclear | Yes | No | No | Yes | Yes | 3 |
| Serrano-Villar 2017 | No | Yes | Yes | Yes | No | No | Yes | Yes | 5 |
| Sinha 2019 | Yes | Yes | Yes | Yes | Yes | Yes | Yes | Yes | 8 |
| Srinivasa 2015 | Yes | Yes | Yes | Yes | Yes | Yes | Yes | Yes | 8 |
| Taylor 2020 | Yes | Yes | Yes | Yes | Yes | Yes | Yes | Yes | 8 |
| Taylor 2020 | No | No | Yes | Yes | Yes | Unclear | Yes | Unclear | 4 |
| Timmons 2014 | Yes | Yes | Yes | Yes | No | No | Yes | Unclear | 5 |
| Wang 2022 | Yes | Yes | Yes | Yes | Yes | Yes | Yes | Yes | 8 |
| Zhang 2019 | Yes | Yes | Unclear | Yes | Yes | Yes | Yes | Yes | 7 |
| Hua 2023 | Yes | Yes | Yes | Yes | Yes | Yes | Yes | Yes | 8 |
| Wang 2023 | Yes | Yes | Yes | Yes | Yes | Yes | Yes | Yes | 8 |
| Kai 2024 | Yes | Yes | Yes | Yes | No | No | Yes | Yes | 6 |
| Peters 204 | Yes | Yes | Yes | Yes | Yes | Yes | Yes | Yes | 8 |
| Sanchez-Conde 2023 | Yes | Yes | Yes | Yes | No | No | Yes | Yes | 6 |
| Martinez-sands 2023 | Yes | Yes | Yes | Yes | No | No | Yes | Yes | 6 |
| Martin 2023 | Yes | Yes | Yes | Yes | No | No | Yes | Yes | 6 |
| Hua 2023 | Yes | Yes | Yes | Yes | Yes | Yes | Yes | Yes | 8 |

1. Were the criteria for inclusion in the sample clearly defined?
2. Were the study subjects and the setting described in detail?
3. Was the exposure measured in a valid and reliable way?
4. Were objective, standard criteria used for measurement of the condition?
5. Were confounding factors identified?
6. Were strategies to deal with confounding factors stated?
7. Were the outcomes measured in a valid and reliable way?
8. Was appropriate statistical analysis used?
