# Supplementary material for: HIV, the gut microbiome and clinical outcomes, a systematic review
Source: PLoS One. 2024 Dec 9;19(12):e0308859. doi: 10.1371/journal.pone.0308859 (PMC11627425; doi:10.1371/journal.pone.0308859)
Supplement: S4 Table — (DOCX) [file pone.0308859.s004.docx]

**S4 Table.** **Quality appraisal result of included studies; Using Joanna Briggs Institute (JBI) quality appraisal checklist** **for Cohort Study designs.**

| **Authors** | **D1** | **D2** | **D3** | **D4** | **D5** | **D6** | **D7** | **D8** | **D9** | **D10** | **Overall** |
| --- | --- | --- | --- | --- | --- | --- | --- | --- | --- | --- | --- |
| Qi 2018 | Yes | Yes | Yes | Yes | Yes | Yes | Yes | Yes | Unclear | Yes | 9 |
| Shan 2018 | Yes | Yes | Yes | Yes | Yes | Yes | Yes | Yes | Unclear | Yes | 9 |
| Kelesidis 2012 | Yes | Yes | Yes | Yes | Yes | No | Yes | Yes | No | Yes | 8 |
| Manner 2013 | Yes | Yes | Yes | Yes | Yes | Unclear | Yes | Yes | No | Yes | 8 |
| Wang 2022 | Yes | Yes | Yes | Yes | not clear | Yes | Yes | Yes | no | Yes | 8 |
| Balagopal 2008 | No | Yes | Yes | Yes | Yes | Unclear | Yes | Yes | Unclear | Yes | 7 |
| Dong 2021 | Yes | Yes | Yes | Yes | Yes | No | Yes | No | Unclear | Yes | 7 |
| Jenabian 2016 | Yes | Yes | Yes | Yes | No | Unclear | Yes | Yes | Unclear | Unclear | 6 |

1. Were the two groups similar and recruited from the same population?
2. Were the exposures measured similarly to assign people to both exposed and unexposed groups?
3. Was the exposure measured in a valid and reliable way?
4. Were confounding factors identified?
5. Were strategies to deal with confounding factors stated?
6. Were the groups/participants free of the outcome at the start of the study (or at the moment of exposure)?
7. Were the outcomes measured in a valid and reliable way?
8. Was the follow up time reported and sufficient to be long enough for outcomes to occur?
9. Were strategies to address incomplete follow up utilized?
10. Was appropriate statistical analysis used?
