# Supplementary material for: HIV, the gut microbiome and clinical outcomes, a systematic review
Source: PLoS One. 2024 Dec 9;19(12):e0308859. doi: 10.1371/journal.pone.0308859 (PMC11627425; doi:10.1371/journal.pone.0308859)
Supplement: S5 Table — (DOCX) [file pone.0308859.s005.docx]

**S5 Table. Quality appraisal result of included studies; Using Joanna Briggs Institute (JBI) quality appraisal checklist for Case-Control Study designs.**

| **Authors** | **D1** | **D2** | **D3** | **D4** | **D5** | **D6** | **D7** | **D8** | **D9** | **D10** | **Overall score** |
| --- | --- | --- | --- | --- | --- | --- | --- | --- | --- | --- | --- |
| ElliottMiller 2016 | Yes | Yes | Yes | Yes | Yes | Yes | Yes | Yes | No | Yes | 9 |
| Gelpi 2019 | Yes | Yes | Yes | Yes | Yes | Yes | Yes | Yes | Yes | Yes | 10 |
| Gelpi 2020 | Yes | Yes | Yes | Yes | Yes | Yes | Yes | Yes | Unclear | Yes | 9 |
| Haissman 2016 | Yes | Yes | Yes | Yes | Yes | Yes | No | Yes | Yes | Yes | 9 |
| Marks 2013 | Yes | Yes | Yes | Yes | Yes | Yes | Yes | Yes | Yes | Yes | 10 |
| Maurice 2019 | Yes | Yes | Yes | Yes | Yes | Yes | Yes | Yes | Yes | Yes | 10 |
| Sinha 2015 | Yes | Yes | Yes | Yes | Yes | Yes | Yes | Yes | Unclear | Yes | 9 |
| Sinha 2019 | Yes | Yes | Yes | Yes | Yes | Yes | Yes | Yes | Yes | Yes | 10 |
| Villanueva-Millán 2019 | yes | yes | yes | yes | yes | yes | yes | yes | unclear | Yes | 9 |
| Ellis, 2022 | Yes | Unclear | Yes | Yes | Yes | Yes | Yes | Yes | Yes | Yes | 9 |

1. Were the groups comparable other than the presence of disease in cases or the absence of disease in controls?
2. Were cases and controls matched appropriately?
3. Were the same criteria used for identification of cases and controls?
4. Was exposure measured in a standard, valid and reliable way?
5. Was exposure measured in the same way for cases and controls?
6. Were confounding factors identified?
7. Were strategies to deal with confounding factors stated?
8. Were outcomes assessed in a standard, valid and reliable way for cases and controls?
9. Was the exposure period of interest long enough to be meaningful?
10. Was appropriate statistical analysis used?
