# Supplementary material for: HIV, the gut microbiome and clinical outcomes, a systematic review
Source: PLoS One. 2024 Dec 9;19(12):e0308859. doi: 10.1371/journal.pone.0308859 (PMC11627425; doi:10.1371/journal.pone.0308859)
Supplement: S6 Table — (DOCX) [file pone.0308859.s006.docx]

**S6 Table.**  **Quality appraisal result of included studies; Using Joanna Briggs Institute (JBI) quality appraisal checklist for Randomised Control Study designs.**

| **Authors** | **D1** | **D2** | **D3** | **D4** | **D5** | **D6** | **D7** | **D8** | **D9** | **D10** | **D11** | **D12** | **Score** |
| --- | --- | --- | --- | --- | --- | --- | --- | --- | --- | --- | --- | --- | --- |
| Dillon 2021 | Yes | No | Yes | No | No | No | Yes | Unclear | Yes | Yes | Yes | Yes | 6 |
| Dirajlal-Fargo 2019 | Yes | No | Yes | No | No | No | Yes | Unclear | Yes | Yes | Yes | Yes | 6 |
| Jinato 2020 | Unclear | Unclear | Yes | Unclear | Unclear | No | Yes | No | No | Yes | Yes | Yes | 4 |
| Timmons 2014 | Unclear | No | Yes | No | No | No | Yes | Unclear | Yes | Yes | Yes | Unclear | 4 |

1. Was true randomization used for assignment of participants to treatment groups?
2. Was allocation to treatment groups concealed?
3. Were treatment groups similar at the baseline
4. Were participants blind to treatment assignment?
5. Were those delivering treatment blind to treatment assignment?
6. Were outcomes assessors blind to treatment assignment?
7. Were treatment groups treated identically other than the intervention of interest?
8. Was follow up complete and if not, were differences between groups in terms of their follow up adequately described and analysed?
9. Were participants analysed in the groups to which they were randomized?
10. Were outcomes measured in the same way for treatment groups?
11. Were outcomes measured in a reliable way?
12. Was appropriate statistical analysis used?
